# Spokewise iridotomy combined with Descemet stripping automated endothelial keratoplasty in iridocorneal endothelial syndrome

**DOI:** 10.3389/fmed.2023.1187009

**Published:** 2023-07-06

**Authors:** Jiaxin Zhang, Rongmei Peng, Gege Xiao, Minshu Wang, Jing Hong

**Affiliations:** ^1^Department of Ophthalmology, Peking University Third Hospital, Beijing, China; ^2^Beijing Key Laboratory of Restoration of Damaged Ocular Nerve, Peking University Third Hospital, Beijing, China

**Keywords:** spokewise iridotomy, iridocorneal endothelial syndrome, Descemet stripping automated endothelial keratoplasty (DSAEK), secondary glaucoma, graft survival

## Abstract

**Purpose:**

Iridocorneal endothelial (ICE) syndrome is a progressive anterior segment disorder that can be tricky to treat. Keratoplasty is commonly used to treat corneal edema in ICE syndrome. However, glaucoma is an important risk factor affecting graft survival. To address this question, we designed a retrospective cohort study to evaluate the effect of Spokewise Iridotomy (SI) on Descemet Stripping Automated Endothelial Keratoplasty (DSAEK) Grafts in Iridocorneal Endothelial (ICE) Syndrome.

**Methods:**

This was a retrospective cohort study. A total of 29 patients were included; 31 eyes with ICE syndrome underwent DSAEK at Peking University Third Hospital between June 2015 and June 2022, including 11 eyes with combined SI during DSAEK. The aim was to explore the effect of SI on vision, glaucoma control, complications, peripheral anterior synechiae recurrence, endothelial cell count, and graft survival.

**Results:**

The median follow-up time was 30.83 months (mo.) in the SI+Endothelial Keratoplasty (EK) group and 6.17 mo in the EK group. The 2-year cumulative survival rate of grafts in the SI+EK group was 100%, compared with the 6-month and 1-year cumulative survival rates of 80.2 and 63.2%, respectively, in the EK group (*p* = 0.043). The SI+EK group had a lower incidence of immediate postoperative complications (*p* = 0.005), fewer postoperative anti-glaucoma medications (AGMs) (*p* = 0.029), smaller peripheral anterior synechiae recurrence (*p* = 0.001), and significant visual acuity improvement (*p* < 0.05). More AGMs were used in failed grafts (*p* = 0.002).

**Conclusion:**

SI can help control intraocular pressure, improve visual acuity, and increase graft survival after DSAEK in ICE syndrome patients.

## 1. Introduction

Iridocorneal endothelial (ICE) syndrome is a rare anterior segment disease characterized by abnormal proliferation and migration of corneal endothelial cells. Typical clinical manifestations include iris atrophy, ectopic pupil, peripheral anterior synechiae (PAS), secondary glaucoma, and corneal edema. The disease progresses with time, with unclear etiology and pathophysiology. Therefore, the clinical management of ICE syndrome is mainly symptomatic to deal with corneal edema and glaucoma, which are the most common causes of vision loss in this disease (1–3).

A recent large retrospective case analysis shows that corneal edema occurs in ~56% of patients, and 14% require keratoplasty (3). Penetrating keratoplasty (PK) can significantly improve visual acuity; however, the long-term outcomes were heterogeneous in different studies, with high allograft rejection and the need for multiple corneal transplants (4–7). Recently, endothelial keratoplasty (EK) has become a preferred option for corneal endothelial disease. Similarly, there is obvious vision improvement in the short-term vision of Descemet stripping automated endothelial keratoplasty (DSAEK), but it is difficult to maintain (8–11). The proportion of failed grafts varies in different studies, ranging from 27 to 77%, including a study by our center showing that the average graft survival time was only 23.4 months (10–13). Graft survival seems to be similar between PK and EK (11, 14). Descemet membrane endothelial keratoplasty (DMEK) is a third-generation corneal endothelial transplantation technology proven to provide better clinical outcomes in ICE syndrome (1, 15–17).

Approximately 73% of patients with ICE syndrome have secondary glaucoma, and 50% require surgical intervention (3). Glaucoma control after keratoplasty is crucial for graft survival (18–20), especially in ICE syndrome (12, 13). However, glaucoma surgery has a double-edged sword effect in keratoplasty; although it helps control intraocular pressure (IOP) (11), it is also an independent risk factor for cell loss after keratoplasty, especially in glaucoma drainage implant (GDI) (11, 19, 20). Peripheral iridectomy is a procedure used to treat primary angle closure, and it uses a laser to create a hole in the iris, allowing aqueous humor to flow directly from the posterior chamber to the anterior chamber, eliminating pupillary block and widening the angle of the chamber. Similarly, we proposed a novel spokewise iridotomy (SI) in ICE syndrome and explored its effect on postoperative IOP control and graft survival.

## 2. Methods

### 2.1. Subjects

A retrospective analysis was performed based on an electronic database to retrieve demographic, surgical, and follow-up data on all patients with ICE syndrome who underwent DSAEK at Peking University Third Hospital between June 2015 and June 2022. Patients with at least 1-month follow-up were consecutively included, excluding patients with other primary or secondary glaucoma etiologies. The diagnosis of ICE syndrome relies on the presence of at least 2 of the following 3 criteria: (1) corneal edema due to abnormal corneal endothelium or typical hammered silver appearance of the corneal posterior surface; (2) iris atrophy with corectopia, uveal ectropion, and iris holes or nodules, occurring in the same eye with corneal changes; (3) broad peripheral anterior synechiae (PAS) or other iridocorneal adhesions anterior to the Schwalbe line (11, 12). The main outcome measures include intraoperative complications; postoperative complications, including immediate IOP elevation, interlayer effusion, dislocation, and rejection; postoperative IOP and PAS recurrence; postoperative best corrected visual acuity (BCVA); central corneal thickness (CCT); graft thickness (GT); anterior recipient corneal bed thickness (ARCBT); endothelial cell density (ECD); endothelial cell loss (ECL); and graft survival status. All patients provided written informed consent to the use of their medical records. This study was approved by the Peking University Third Hospital Institutional Review Board and adhered to the tenets of the Declaration of Helsinki.

### 2.2. Surgical procedure

**Figure 1:**
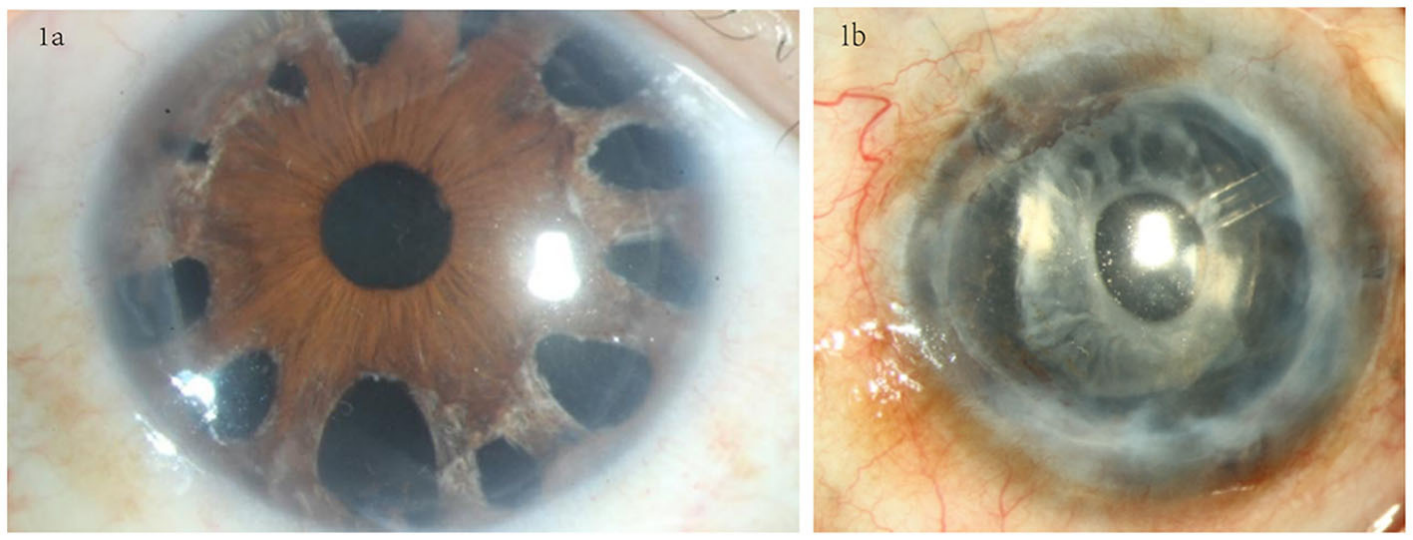
Iris processing in the SI+EK group. **(a)** Spokewise iridotomy in eyes with relatively intact iris structures; iridotomy was performed at the PAS site using a vitreous cutter. **(b)** Total iridectomy was performed in eyes with severe iris atrophy.

The surgery was performed following the standard procedures for DSAEK combined with the suture pull-through insertion technique (13, 21). The graft diameter was determined according to each patient's specific requirements: between 7.5 and 8.25 mm. Additional intraoperative procedures included phacoemulsification and synechiolysis in some cases. In eyes (*n* = 10) with extensive PAS and a relatively intact iris structure, a 23G vitreous cutter was used to perform spokewise iridotomy from the root of the iris within the range of PAS, moderate negative pressure (350 mmHg) and cutting frequency (800 times/min) were used, the incision hole was open 1–2 mm diameter, the diameter of iris root was between 0.5 and 3 mm, and the root incision should be spaced 0.5–1 h apart (Figure 1a); in eyes (*n* = 1) with extensive PAS and iris atrophy, a total iridectomy was performed from the root of the iris (Figure 1b).

### 2.3. Postoperative management

Postoperative management is the same between the two groups. The patients maintain the supine position for 4 h postoperatively, and the patients' eyes were evaluated on the night of surgery-day, 1 day, 1 week, 1 month, 3 months, 6 months, and 12 months after surgery. Topical antibiotics, topical corticosteroids, cyclosporine eye drops, and artificial tears were administered 4 times a day for 1 month. The dose was tapered as clinically indicated, and prednisolone acetate 1.0% was maintained once daily after 12 months.

### 2.4. Outcome measures

Collected data include gender, age, glaucoma, previous surgical history, preoperative BCVA, IOP, AGMs, PAS, ECD, CCT, donor size and ECD, combined surgery, postoperative BCVA, IOP, AGMs, PAS, surgery, and graft status. BCVA was assessed using the Snellen chart and then converted to logMAR values for statistical data analysis. IOP was measured using a Goldmann applanation tonometer or icare tonometer. Anterior segment and graft survival was assessed using slit-lamp biomicroscopy. CCT was assessed using the anterior segment-optical coherence tomography (AS-OCT) system with LASIK flap tool software. The range of PAS was judged based on the results of AS-OCT, ultrasonic biomicroscopy (UBM), gonioscopy, and slit-lamp microscopy. For patients in the SI+EK group, only PAS without an iris incision was recorded because the degree of PAS at the site of iris incision was less severe and did not affect the flow of aqueous humor (Supplementary Figure 1). Preoperative donor ECD was obtained from eye bank donor documentation, as counted with a specular microscope. Postoperative ECD was measured with a ConfoScan microscope (13). Data were excluded when ECD was unclear. The diagnostic criteria for graft failure were irreversible cornea edema with normal IOP, unrelieved with steroids, and the first time observed irreversible cornea edema recorded as the time of graft failure. Primary graft failure was defined as unimproved cornea edema postoperatively; secondary graft failure was defined as irreversible, progressive corneal edema after an initial period of corneal clarity after transplantation (12, 13).

### 2.5. Statistical analysis

Statistical analysis was performed using the SPSS software version 27.0. A Shapiro–Wilk test was used for continuous variables to assess the normality. Normally distributed variables were reported as the mean ± SD, and non-normally distributed variables were reported as the median (25th percentile and 75th percentile). Categorical variables are described using frequency distributions and percentage values. A two-sided two-sample *t*-test was used to compare normally distributed data, and a Wilcoxon rank-sum test was used for non-normally distributed data. Differences in longitudinal data were analyzed using one-way ANOVA. Categorical variables were analyzed using χ^2^-analysis, and Fisher's exact test for small sample data (*n* < 5). Kaplan–Meier survival analysis was used to evaluate the graft survival rate at various times, and potential risk factors for graft failure were evaluated using a log-rank test and Cox proportional hazard regression. Statistical significance was defined as *p* < 0.05.

## 3. Results

### 3.1. Demographic and medical history

A total of 29 patients (31 eyes) were consecutively included; 11 (35.5%) eyes were combined with SI and 20 (64.5%) eyes were not combined. The median postoperative follow-up time was 30.83 months (range 2–84 months) and 6.17 months (range 1–67 months), with no significant difference (*p* = 0.117). Five and six patients had an antiglaucoma surgery history in the SI+EK group and EK group, respectively (Supplementary Table 2). The detailed data of each patient are shown in Supplementary Table 1.

### 3.2. Preoperative and surgical details

The preoperative mean BCVA in the two groups was 1.43 ± 0.46 logMAR and 1.23 ± 0.48 logMAR, median IOP was 18.00 mmHg (IQR 15.10–20.00 mmHg) and 16.00 mmHg (IQR 12.50–21.75 mmHg), AGMs was 0 (IQR 0–2) and 0 (IQR 0–1.75), and median PAS range was 12 and 9 o'clock, with no significant difference (*p* > 0.05). Graft size was 8.00 mm (IQR 7.50–8.00) and 8.00 mm (IQR 8.00–8.00), donor ECD was 3,053 cells/mm^2^ (IQR 3,000–3,333) and 3,097.5 cells/mm^2^ (IQR 3,000–3,315.75). In the SI+EK group, five eyes combined cataract surgery and one eye underwent total iridectomy due to severe iris atrophy; eight eyes in the EK group combined cataract surgery. In the SI+EK group, the median number of iris incisions was 4.50 (IQR 3.00–6.75), and the average maximum interval of iris incisions was 3.05 ± 1.97 (Supplementary Table 3).

### 3.3. Complications

On the night of surgery, 10 eyes (50.0%) in the EK group developed pupillary block with significantly increased IOP. Anterior chamber puncture was required to release some gas and thus reduce the IOP in these eyes. However, this complication did not occur in the SI+EK group. The difference is statistically significant (*p* = 0.005). The mean IOP on the first postoperative day was higher in the EK group than in the SI+EK group (28.36 ± 12.80 mmHg vs. 15.21 ± 6.24 mmHg, *p* = 0.001). Both groups did not have a dislocation, interlayer effusion, or rejection. Three eyes in the EK group underwent surgery at 2, 3, and 5 months after keratoplasty due to the uncontrollable IOP (Supplementary Table 1). Post-op AGMs were higher in the EK group than in the SI+EK group [1.5 (IQR 0–3) vs. 0 (IQR 0–1), *p* = 0.029], and the increase of AGMs was higher in the EK group [0.5 (IQR 0–3) vs. 0 (IQR −1–0), *p* = 0.039]. Two eyes in the SI+EK group underwent synechiolysis for obvious local PAS recurrence, and one eye in the EK group underwent EK+synechiolysis. The recurrence range of PAS was lower in the SI+EK group (2.44 ± 1.67 vs. 6.09 ± 3.59, *p* = 0.012), and the range of PAS reduction was higher in the SI+EK group [8 (IQR 8–11) vs. 2.27 ± 4.38, *p* = 0.001] (Table 1).

**Table 1:** Complications in the different surgical groups.

	**SI+EK (*n* = 11)**	**EK (*n* = 20)**	***P*-value**
Pupillary block high IOP (*n*, %)			0.005
Yes	0 (0%)	10 (50.0%)	
No	11 (100%)	10 (50.0%)	
IOP on the first-day post-op (mmHg)	15.21 ± 6.24	28.36 ± 12.80	0.001
Post-op AGMs (*n*)^a^	1 (0, 1)	1.5 (0, 3)	0.029
Post-op AGMs increase (*n*)^a^	0 (−1, 0)	0.5 (0, 2)	0.039
Post-op antiglaucoma surgery (*n*, %)			0.535
Yes	0 (0%)	3 (15.0%)	
No	11 (100%)	17 (85.0%)	
Post-op PAS range (*n*)^b^	2.44 ± 1.67	6.09 ± 3.59	0.012
Post-op PAS range decrease (*n*)^b^	8 (8, 11)	2.27 ± 4.38	0.001
Post-op synechiolysis (*n*, %)			0.281
Yes	2 (18.2%)	1 (5.0%)	
No	9 (81.8%)	19 (95.0%)	

SI, spokewise iridotomy; EK, endothelial keratoplasty; IOP, intraocular pressure; post-op, postoperation; AGMs, antiglaucoma medications; PAS, peripheral anterior synechiae.

a Three eyes requiring surgery were calculated according to the number of AGMs used at the last follow-up time before surgery.

b Three eyes that underwent synchiolysis were calculated according to the data at the last follow-up time. Available data in 9 eyes from the SI+EK group and 11 from the SI+EK group.

### 3.4. Follow-up data

BCVA was significantly improved after DSAEK, which was better in the SI+EK group. The difference was most significant 6 months after surgery [0.10 (IQR 0.10–0.40) vs. 0.91 (IQR 0.23–1.85), *p* = 0.012] (Supplementary Figure 2). Graft thickness tended to be thicker in the EK group, but the difference was only statistically significant at 1 month postoperatively (81.00 ± 32.98 um vs. 128.62 ± 65.91 um, *p* = 0.045). During the follow-up, ECL mainly occurred at 1 month after surgery, and there was little change in ECD until 12 months (one-way ANOVA, *p* = 0.746 and 0.609). ECD at 1, 6, and 12 months was 1,352.14 ± 593.70 cells/mm^2^ vs. 1,334.38 ± 578.61 cells/mm^2^, 1,168.86 ± 602.55 cells/mm^2^ vs. 1,036.83 ± 480.50 cells/mm^2^, and 1,308.80 ± 503.20 cells/mm^2^ vs. 1,463.00 ± 610.94 cells/mm^2^, respectively. There was no significant difference in ARCBT, CCT, ECD, and ECL between SI+EK and EK groups (Table 2).

**Table 2:** Follow-up data in the different surgical groups.

		**Follow-up visit**				
		**1 week**	**1 month**	**3 months**	**6 months**	**12 months**
BCVA^a^	SI+EK	0.56 ± 0.32	0.48 ± 0.24	0.35 ± 0.28	0.10 (0.05, 0.40)	0.16 ± 0.15
	EK	1.07 ± 0.57	0.92 ± 0.55	0.92 ± 0.58	0.91 (0.23, 1.85)	-
	P	0.025	0.036	0.014	0.012	-
CCT (um)^b^	SI+EK	728.78 ± 102.30	627.25 ± 65.28	603.83 ± 62.18	589.75 ± 57.84	567.00 ± 69.51
	EK	711.25 ± 120.43	638.77 ± 94.14	633.50 (559.75, 663.75)	691.29 ± 139.28	601.50 ± 30.41
	P	0.717	0.765	0.699	0.061	0.570
GT (um)^b^	SI+EK	125 (95.0, 182.5)	81.00 ± 32.98	93.17 ± 29.50	79.00 ± 30.38	67.33 ± 38.80
	EK	135.63 ± 79.78	128.62 ± 65.91	106.43 ± 31.88	113.00 (65.00, 191.00)	114.50 ± 67.18
	P	0.821	0.045	0.456	0.110	0.378
ARCBT (um)^b^	SI+EK	556.22 ± 150.19	533.00 ± 54.64	510.67 ± 49.89	484.14 ± 60.96	499.67 ± 38.73
	EK	547.50 (515.25, 619.25)	510.15 ± 45.60	503.14 ± 29.37	537.14 ± 62.86	487.00 ± 36.77
	P	0.713	0.331	0.742	0.218	0.740
ECD (cells/mm^2^)^c^	SI+EK	-	1,352.14 ± 593.70	1,475.86 ± 534.30	1,168.86 ± 602.55	1,308.80 ± 503.20
	EK	-	1,334.38 ± 578.61	1,421.17 ± 556.45	1,036.83 ± 480.50	-
	P	-	0.954	0.906	0.675	-
ECL (%)^c^	SI+EK	-	56.69 (43.38, 79.76)	52.73 ± 17.75	62.27 ± 19.36	58.48 ± 18.66
	EK	-	56.99 ± 20.02	54.92 ± 18.66	66.28 ± 16.88	-
	P	-	0.817	0.833	0.700	-

SI, spokewise iridotomy; EK, endothelial keratoplasty; BCVA, best corrected visual acuity; CCT, central corneal thickness; GT, graft thickness; ARCBT, anterior recipient corneal bed thickness; ECD, endothelial cell density; ECL, endothelial cell loss.

a Six eyes with other vision-affecting causes were excluded (two in the SI+EK group and four in the EK group), and available data of EK at 12 months was <50%.

b Available data were three eyes from the SI+EK group and two from the EK group at 12 months.

c Unclear ECD was excluded, available data were seven eyes from the SI+EK group and eight from the EK group at 1 month, seven and six eyes at 3 months, seven and six eyes at 6 months, and five eyes in the SI+EK group at 12 months.

### 3.5. Graft survival and risk factors

**Figure 2:**
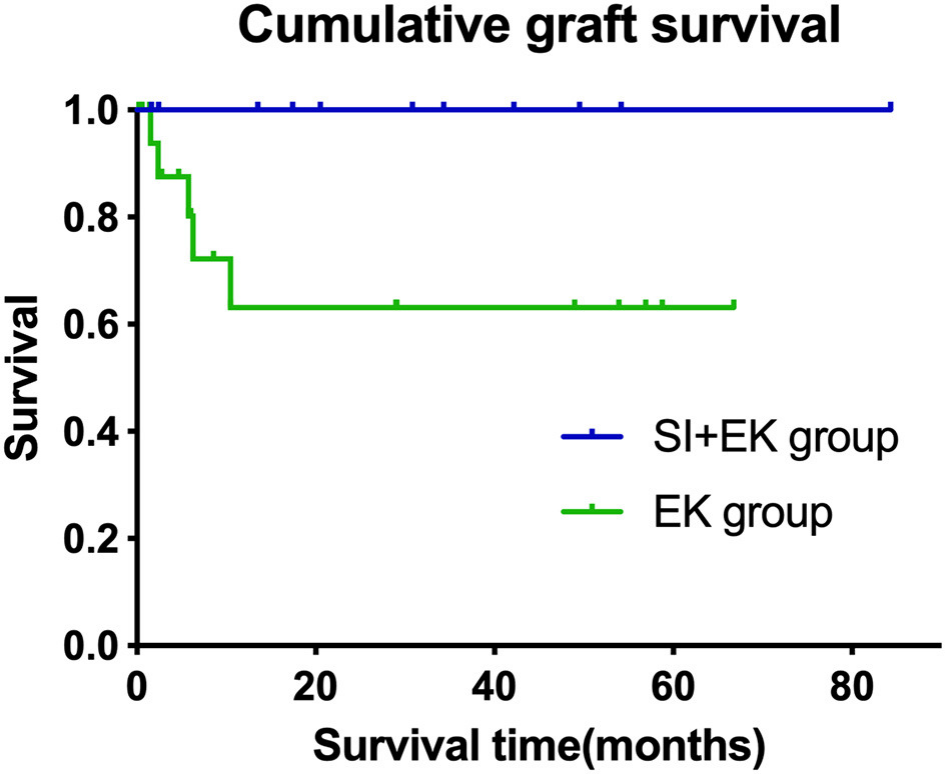
Cumulative graft survival in the different surgical groups. Kaplan–Meier survival curve, demonstrating cumulative survival over time in months. The cumulative survival rate of SI+EK at 12 and 24 months was 100%, compared with the survival rate of EK at 3, 6, and 12 months, which was 87.5, 80.2, and 63.2%, respectively (log-rank test, *p* = 0.043).

At a mean follow-up time of 5.29 ± 3.57 months, irreversible corneal edema occurred in 5 of 31 grafts. All five failure grafts were diagnosed as secondary graft failure due to endothelial cell failure (three were operated on by surgeon 1 and two by surgeon 2). Kaplan–Meier analysis showed that there was a difference between the estimated cumulative graft survival rates of the SI+EK group and the EK group (log-rank test, *p* = 0.043), the estimated survival rate of SI+EK at 12 and 24 months was 100%, the estimated survival rate of EK at 3, 6, and 12 months were 87.5, 80.2, and 63.2%, respectively (Figure 2).

Follow-up time was similar in failed and survival grafts. Although the smaller sample size of the failed grafts made it difficult for the results to be statistically significant, we observed that the failed grafts had a higher number of postoperative AGMs (Wilcoxon rank-sum test, *p* = 0.002), a higher proportion of post-op antiglaucoma surgery (log-rank test, *p* = 0.023), and pre-op cataract surgery (log-rank test, *p* = 0.039) (Table 3). No risk factors were identified in Cox proportional hazard regression.

**Table 3:** Analysis of risk factors for graft failure.

	**Failure grafts (*n* = 5)**	**Survival grafts (*n* = 26)**	***P*** **-values**	
			**P1^a^**	**P2^b^**
Age (year)	54.20 ± 13.99	53.92 ± 11.73	0.968	-
Follow-up time (month)	5.29 ± 3.57	27.65 (2.68, 50.61)	0.119	-
Gender (male number, %)	3 (60.0%)	16 (61.5%)	1.000	0.760
Pre-op glaucoma (*n*, %)	4 (80.0%)	15 (57.7%)	0.624	0.440
Pre-op EK history (*n*, %)	2 (40.0%)	5 (19.2%)	0.562	0.428
Pre-op cataract surgery (*n*, %)	4 (80.0%)	7 (26.9%)	0.042	0.039
Pre-op antiglaucoma surgery history (*n*, %)	3 (60.0%)	8 (30.8%)	0.317	0.160
Pre-op AGMs (*n*)	0.80 ± 0.84	0 (0, 2)	0.651	-
Surgery (SI+EK *n*, %)	0 (0%)	11 (42.3%)	0.133	0.043
Combined cataract surgery (*n*, %)	0 (0%)	13 (50.0%)	0.058	0.061
Surgeon (surgeon 1, %)	3 (60.0%)	15(57.7%)	0.875	0.858
Pupillary block high IOP (*n*, %)	3 (60.0%)	7 (26.9%)	0.296	0.061
IOP on the first-day post-op (mmHg)	28.80 ± 8.90	18.50 (13.75, 31.50)	0.101	-
Post-op AGMs (*n*)	3 (2.5, 3.0)	0 (0, 2)	0.002	-
Post-op antiglaucoma surgery (*n*, %)	2 (40.0%)	1 (3.8%)	0.060	0.023
Post-op synechiolysis (*n*, %)	1 (33.3%)	2 (7.7%)	0.422	0.519

a p-value of difference analysis between two groups by χ^2^-analysis or t-test or Wilcoxon rank-sum test.

b p-value of the log-rank test.

## 4. Discussion

We innovatively performed SI as an auxiliary surgery for DSAEK in ICE syndrome, and the clinical outcomes showed that SI could significantly reduce short-term postoperative IOP, reduce the AGMs and PAS recurrence after EK, and improve postoperative BCVA and cumulative survival rate of grafts.

Two grafts in the EK group had graft failure within 6 months of postoperative follow-up; therefore, we did not exclude patients with a follow-up time of <6 months. The follow-up time tended to be shorter in the EK group, which was related to the endpoint event loss.

The presence of multiple iris incisions in the SI+EK group could effectively avoid immediate postoperative complications (pupillary block high IOP requiring release of anterior chamber gas) (*p* = 0.005), avoiding transient IOP increase in ICE syndrome eyes with a fragile IOP accommodation system, which may affect endothelial cell activity and thereby accelerate the onset of secondary graft failure, as shown in our previous study (13). No other postoperative complications were observed in either group, and the lower rejection in our center may be related to shorter postoperative follow-up time and better patient compliance (12, 13). As for the additional side effects brought by SI, careful follow-up showed that only one patient (no. 6) complained of mild outdoor glare and other patients did not complain of discomfort, similar to previous reports (12, 22).

In the long-term follow-up, post-op AGMs and the increase of AGMs were higher in the EK group. The difference in post-op AGMs indicated that SI still played a role in controlling IOP during postoperative follow-up. Nearly half of ICE syndrome patients after DSAEK will have significantly elevated IOP, which requires AGMs increase or antiglaucoma surgery to control (2, 11, 12). This is consistent with the data in this study that 10 eyes (50.0%) in the EK group had significantly higher postoperative IOP (>30 mmHg), compared with one eye (9.1%) in the SI+EK group. The increased IOP after DSAEK may be due to a more crowded anterior chamber after implantation of a 120-um graft in the anterior chamber, the need for long-term use of steroid eye drops, and the short-term postoperative viscoelastic and gas residues. Combined SI during DSAEK may help alleviate these difficulties. In addition, this innovative procedure may also avoid the double-edged sword effect of glaucoma surgery before EK, because SI does not have a device that could be in mechanical contact with the corneal endothelium, like GDI.

UBM or OCT imaging of the anterior segment is a useful tool in corneal edema to detect PAS (2). SI could effectively reduce the range of postoperative PAS recurrence. Although synechiolysis was combined in almost all cases, PAS recurred during the follow-up, consistent with previous reports (16). However, we observed that the iris had filiform adhesion (to a lesser extent) in the position where the iris was undercut, and the recurrent PAS did not affect the flow of the aqueous humor, which may be the key to SI helping to control IOP.

The visual acuity improvement in the SI+EK group was more significant, and the data were statistically significant in the first 6 months. In addition, we noticed that GT was smaller in the SI+EK group, especially at 1 month postoperatively. There was a trend toward smaller CCT in the SI+EK group. These outcomes suggest that the reduction of corneal edema in the SI+EK group was better, accompanied by a better recovery of corneal clarity, resulting in a better improvement in BCVA, which may be related to better postoperative IOP control.

There was no significant difference in ECD and ECL between the two groups, indicating that the protective effect of SI may not be exerted by reducing ECL. Nonetheless, we could believe that the more complex surgical procedure in the SI+EK group did not result in additional perioperative ECL. We compared the EK group in our study with DSEK cases [since there is evidence that DMEK is superior to DSEK in achieving faster visual recovery, better visual outcomes, and lower rates of rejection (23, 24), we did not include DMEK data in this analysis (16, 17)] (Table 4). The follow-up time of this study was relatively short, ECL at the same follow-up time fell between the data of Fajgenbaum and Hollick (10) (graft failure rates were 78, 80, and 83% at 6 months, 1 year, and 2 years, respectively) and the data from the previous report of our center (13) (graft failure rates were 52, 58, and 69% at 6 months, 1 year, and 2 years, respectively) were higher than the data of Mohamed et al. (12) (graft failure rates were 28, 38, and 44% at 6 months, 1 year, and 2 years, respectively). ECL in the perioperative period is mainly due to traumatic factors in the operation. All operations in this study were performed by four different surgeons; the learning curve of the surgeons may be the reason for the discrepancy between the data in this study and the previous data in our unit (13). However, there was no difference in the distribution of surgeons between the two groups, and surgeons were not a risk factor for graft failure. Although the protective effect of SI on the graft was not seen in ECD, the more complex procedure in the SI+EK group did not result in additional cell loss.

**Table 4:** Clinical study of DSAEK/DSEK in ICE syndrome.

**Study**	**Graft (*n*)**	**Year**	**Average follow-up time (range)**	**Pre-op vision (logMAR)**	**Pre-op antiglaucoma surgery**	**Best post-op vision (logMAR)**	**Failed graft**	**ECD at the last follow-up**	**ECL at the last follow-up**
Fajgenbaum et al. (10)	9	2015	25 mo (6–52 m)	-	77%	-	7 (77%)	-	83
Quek et al. (11)	12	2015	48 ± 31.2 mo	-	34%	-	4 (33%)	-	-
Previous report of our center (13)	20	2018	19.0 mo (6–36 m)	1.51 ± 0.55	55%	0.57 ± 0.34	11 (55%)	1,148.2 ± 1,217.8	69.3
Mohamed et al. (12)	52	2022	28.8 mo (6–109 m)	2.78 (0.85–3.00)	37%	0.40	14 (27%)	1,429.5 ± 136.9	56.9
Our study	20	2023	6.17 mo (1–67 m)	1.23 ± 0.48	30%	0.91 (0.23, 1.85)	5 (23.8%)	1,036.8 ± 480.5	66.3

The cumulative survival rates of the SI+EK group at 6 months, 1 year, and 2 years were all 100%. The 3 months, 6 months, and 1-year cumulative survival rates of the EK group were 87.5, 80/2, and 63.2%, respectively. The median survival time could not be calculated due to the small number of failed grafts. Cumulative survival rates of the previous report of our center (13) were 95.0, 85.0, and 53.1% at 6 months, 1 year, and 2 years, respectively, and of Mohamed et al. (12) were 93.6 and 85.6% at 1 and 2 years, respectively. Reviewing the previous reports, we found that the survival rate of the SI+EK group was better than the previous data, and the survival rate of the EK group was within the range of the previous data; moreover, we found it interesting that there appears to be a correlation between graft failure rate and history of glaucoma surgery (Table 4).

The proportion of pre-op cataract surgery is higher in failed grafts, which may partly indicate a more complex history and a slightly more severe degree of glaucoma in the failed grafts. AGMs and the proportion of post-op antiglaucoma surgery are higher in failed grafts, suggesting that these grafts may experience uncontrollable IOP elevations before failure. This is consistent with previous studies finding that glaucoma surgery after keratoplasty is a risk factor for graft failure, suggesting that the IOP of failed grafts may be more difficult to control (8, 11, 13, 18–20, 25, 26).

Since Chandler syndrome is the most severe subtype involving the corneal endothelium, most patients included in this study had Chandler syndrome. All the patients we included in the SI+EK group had Chandler syndrome. The characteristic indicator of this syndrome is that the endothelium and basement membrane extend beyond the peripheral cornea, leading to PAS and refractory glaucoma (27). SI could effectively reduce iris adhesion, although some patients needed synechiolysis again for PAS recurrence (2/11). While AGMs did not reduce, they did not increase after 12 months of follow-up, with no patient needing anti-glaucoma surgery either. This suggests that although SI did not reduce the severity of glaucoma, it may control the progression of glaucoma. The possible reason is that SI removes the abnormal proliferation membrane at the root of the iris. The elastic fibers of the remaining membrane tissue shrink. The membrane tissue originally attached to the trabecular meshwork leaves the trabecular meshwork. The IOP reduces following the function of the trabecular meshwork recovery.

In conclusion, the short-term clinical outcomes of SI as an auxiliary surgery for DSAEK in ICES syndrome are favorable. Compared with Chaurasia et al. (22), we expanded the scope of patients undergoing iridectomy and attempted spokewise iridotomy for patients with intact iris structures but broad PAS. The advantages of this operation are (1) to avoid the occurrence of immediate IOP increase after surgery; (2) effectively reduces the range of PAS recurrence and plays an auxiliary role in regulating IOP, therefore, playing a protective role in the survival of the graft; (3) excision of part of the iris at the adhesion site can more thoroughly remove the abnormal endothelial membrane covering the angle and iris surface, which may play a role in delaying the progression of the disease (28).

This study was limited to its retrospective nature, small sample size, data missing, and short follow-up time in the EK group, mainly due to the rarity of ICE syndrome. Nonetheless, we found that SI exerted a protective effect on graft survival. Long-term follow-up data are needed to observe the effect of SI in ICE syndrome in the future.

## Data availability statement

The original contributions presented in the study are included in the article/Supplementary material, further inquiries can be directed to the corresponding author.

## Ethics statement

The studies involving human participants were reviewed and approved by Peking University Third Hospital Institutional Review Board. The patients/participants provided their written informed consent to participate in this study.

## Author contributions

Conceived and designed the study: JH. Surgery treatment and clinical follow-up: RP, GX, MW, and JH. Collected the data: JZ and MW. Wrote the paper: JZ and RP. All authors contributed to the article and approved the submitted version.
