# Supplementary material for: Spokewise iridotomy combined with Descemet stripping automated endothelial keratoplasty in iridocorneal endothelial syndrome
Source: Front Med (Lausanne). 2023 Jul 6;10:1187009. doi: 10.3389/fmed.2023.1187009 (PMC10357380; doi:10.3389/fmed.2023.1187009)
Supplement: Supplementary file 1 [file Data_Sheet_1.pdf]

Supplementary table 1

Preoperative, intraoperative and postoperative details for SI+EK group												
**Case**	**Age/ gender**	**Eye**	**Pre-op BCVA (Snellen)**	**Pre-op clinical findings**	**Prior glaucoma (Y/N)**	**Prior AGMs**	**Prior surgical history**	**Surgical procedures**	**Post-op BCVA (Snellen)**	**Post-op AGMs**	**Post-op events**	**Follow-up (months)**
1	67/M	OS	FC/50cm	Iris atrophy, corectopia, PAS at 12 to 9 o'clock, bleb superiorly	Yes	1	-	DSAEK, synechiolysis, phaco/PCIOL, peripheral iridotomy at 1 2 4 6 7 10 o'clock	20/400	1	Vision doesn't improve due to maculopathy	17
2	49/M	OS	HM/50cm	Iris atrophy, corectopia, broad PAS, bleb superiorly	Yes	2	EK, Trab, GDD, phaco/PCIOL	DSAEK, iridectomy	HM/15cm	2	Vision doesn't improve due to glaucomatous optic neuropathy	34
3	63/M	OD	20/1000	Broad PAS, bleb superiorly	Yes	0	Anti-glaucoma surgery(type unknown)	DSAEK, synechiolysis, phaco/PCIOL, peripheral iridotomy at 2 7 9 o'clock	20/40	0	-	2

Case	Age/ gender	Eye	Pre-op BCVA (Snellen)	Pre-op clinical findings	Prior glaucoma (Y/N)	Prior AGMs	Prior surgical history	Surgical procedures	Post-op BCVA (Snellen)	Post-op AGMs	Post-op events	Follow-up (months)
4	36/M	OD	20/80	Iris atrophy, corectopia, broad PAS, bleb superiorly	Yes	2	-	DSAEK, synechiolysis, peripheral iridotomy at each hour with 12 incisions	20/25	0	Synechiolysis was performed at 21-month due to PAS at 7 to 12 o’clock	42
5	73/F	OS	20/333	Iris atrophy, corectopia, bleb nasally, broad PAS, iris root incision at 10 and 12 o’clock	Yes	1	EK, Trab, vitrectomy, epiretinal macular membrane exfoliation phaco/PCIOL	DSAEK, synechiolysis, peripheral iridotomy at 2 6 7 8 o’clock	20/133	0	-	14
6	79/M	OD	FC/30cm	Iris atrophy, corectopia, broad PAS, iris root incision at 12 o’clock	Yes	0	EK, phaco/PCIOL, Trab	DSAEK, synechiolysis, peripheral iridotomy at 5 6 7 8 9 10 o’clock	20/50	0	Followed up on call since 9-month	31

Case	Age/ gender	Eye	Pre-op BCVA (Snellen)	Pre-op clinical findings	Prior glaucoma (Y/N)	Prior AGMs	Prior surgical history	Surgical procedures	Post-op BCVA (Snellen)	Post-op AGMs	Post-op events	Follow-up (months)
7	40/F	OD	20/250	Broad PAS	Yes	0	-	DSAEK, synechiolysis, peripheral iridotomy at 1 2 4 6 7 8 9 11 12 o'clock	20/20	0	Followed up on call since 30-month	50
8	44/M	OD	FC/20cm	Iris root incision at 11 o'clock, corectopia, PAS at 1 to 6 and 10 to 11 o'clock, bleb superiorly	Yes	0	EK, peripheral iridotomy	DSAEK, synechiolysis, peripheral iridotomy at 3 6 9 o'clock	20/80	2	-	2
9	55/F	OS	FC/15cm	Iris atrophy, corectopia, broad PAS, bleb superiorly	No	0	-	DSAEK, phaco/PCIOL, synechiolysis, iridotomy at 3 6 7 9 o'clock	20/20	0	-	21

Case	Age/ gender	Eye	Pre-op BCVA (Snellen)	Pre-op clinical findings	Prior glaucoma (Y/N)	Prior AGMs	Prior surgical history	Surgical procedures	Post-op BCVA (Snellen)	Post-op AGMs	Post-op events	Follow-up (months)
10	58/F	OD	20/200	Iris atrophy, broad PAS, bleb superiorly	Yes	3		DSAEK, phaco/PCIOL, synechiolysis, iridotomy at 4 6 9 o'clock	20/33	0	Synechiolysis was performed at 3-month due to PAS, followed up on call since 12-month	54
11	53/M	OS	20/1000	Iris atrophy, corectopia, broad PAS, bleb superiorly	No	0	-	DSAEK, phaco/PCIOL, synechiolysis, iridotomy at 5 6 10 o'clock	/	0	Followed up on call since 24-month.	84

Supplementary Table 1-1. Preoperative, intraoperative and postoperative details for each case in SI+EK group. SI, spokewise iridotomy; EK, endothelial keratoplasty; Pre-op, preoperative; Post-op, postoperative; BCVA, best corrected visual acuity; AGM, antiglaucoma medication; PAS, peripheral anterior synechiae; Trab, trabeculectomy; PCIOL, posterior chamber intraocular lens; DSAEK, descemet stripping automated endothelial keratoplasty; IOP, intraocular pressure.

Preoperative and postoperative photographs for SI+EK group		
**Case**	**Pre-op**	**Post-op**
01	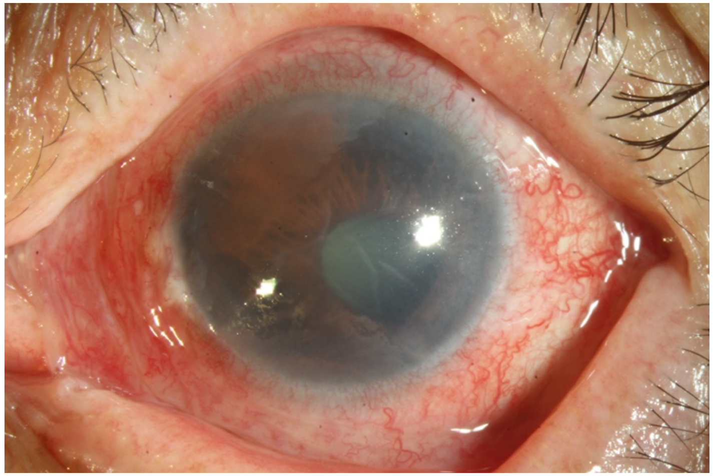	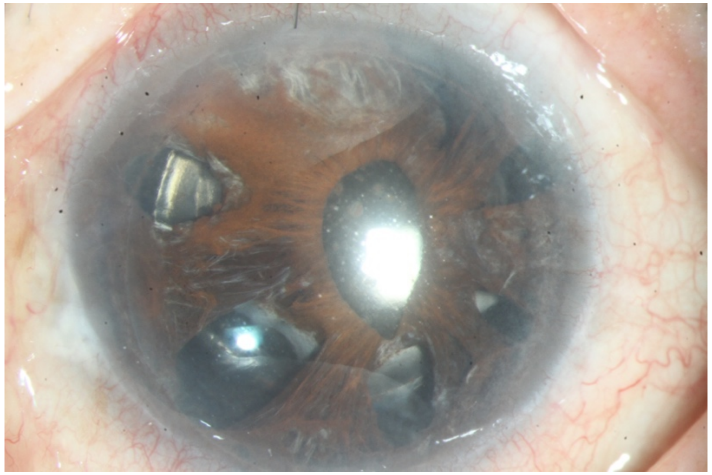
02	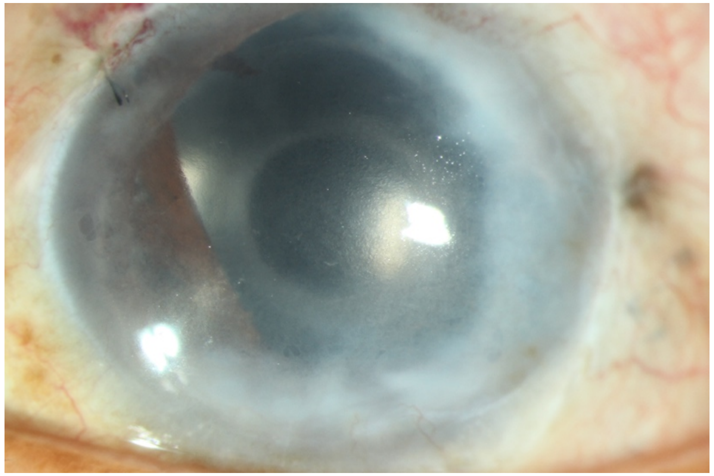	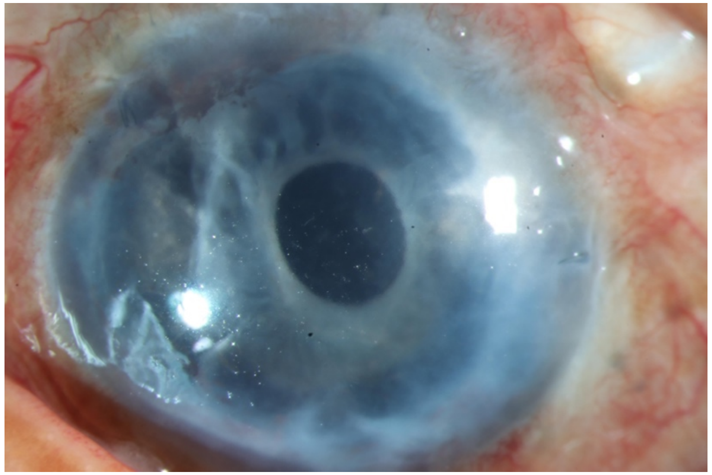

Case	Pre-op	Post-op
03	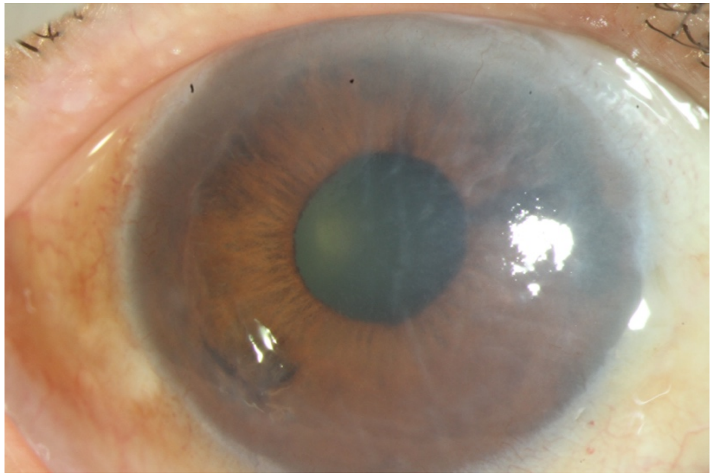	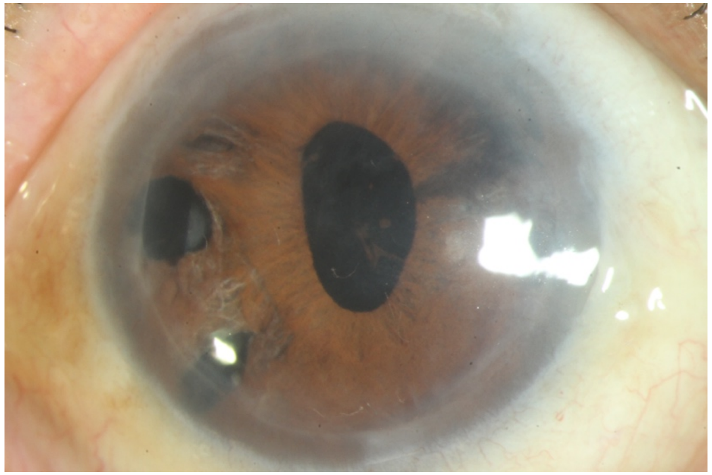
04	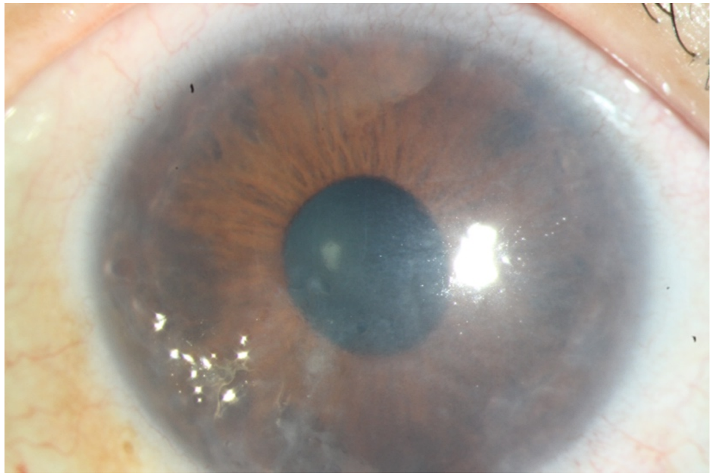	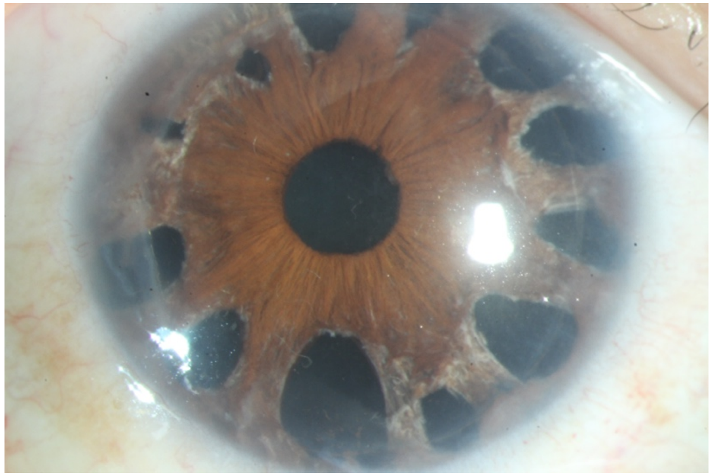

Case	Pre-op	Post-op
05	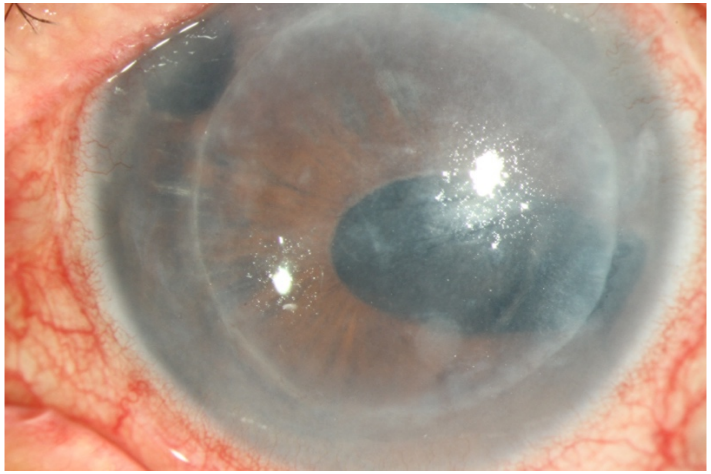	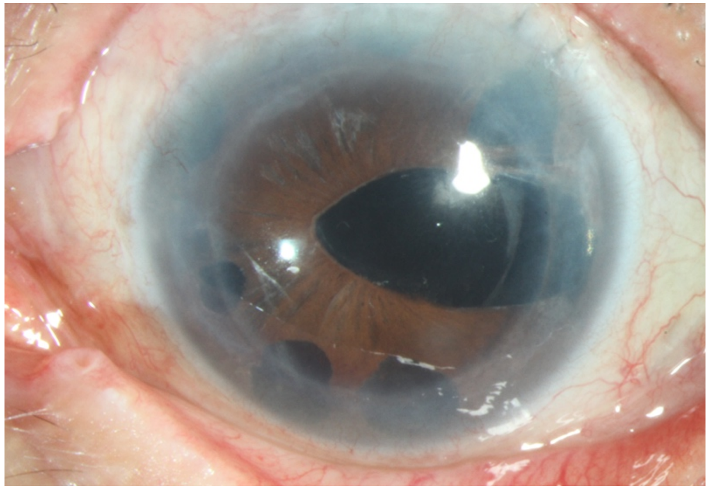
06	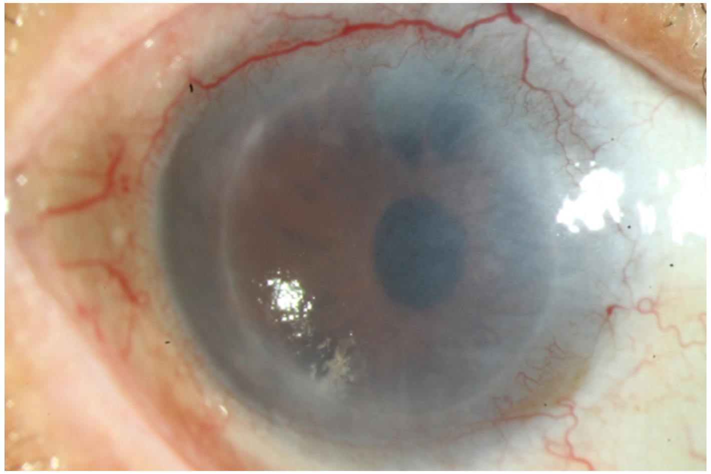	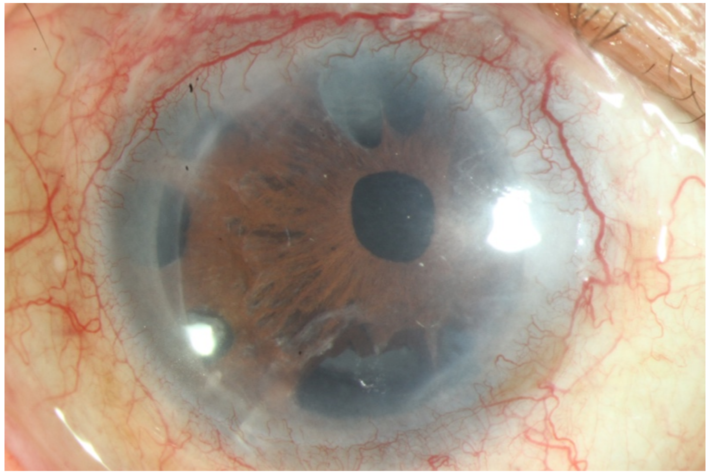

Case	Pre-op	Post-op
07	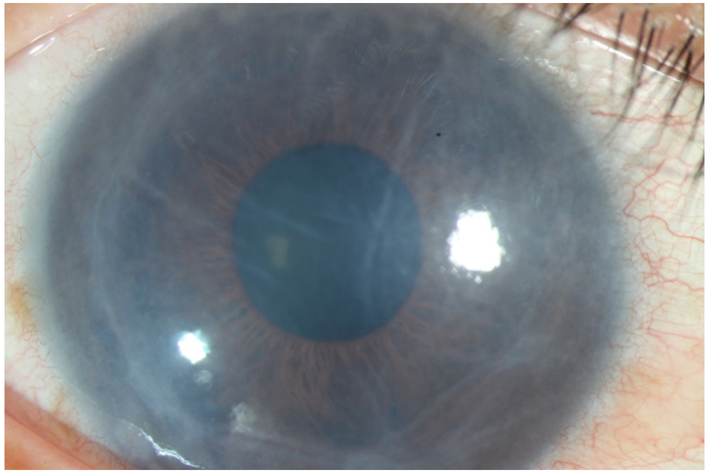	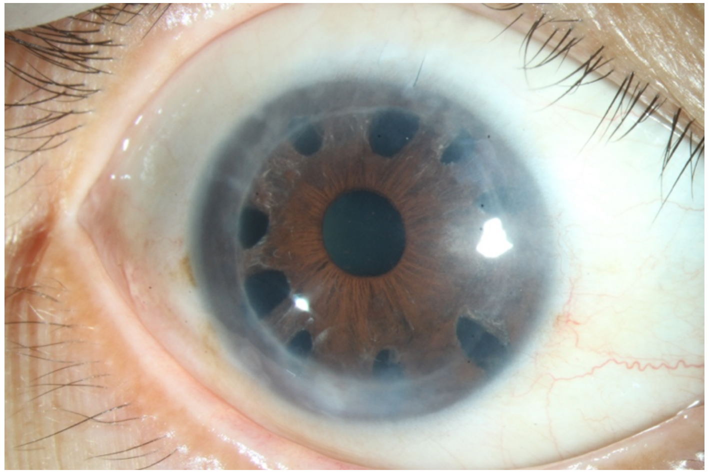
08	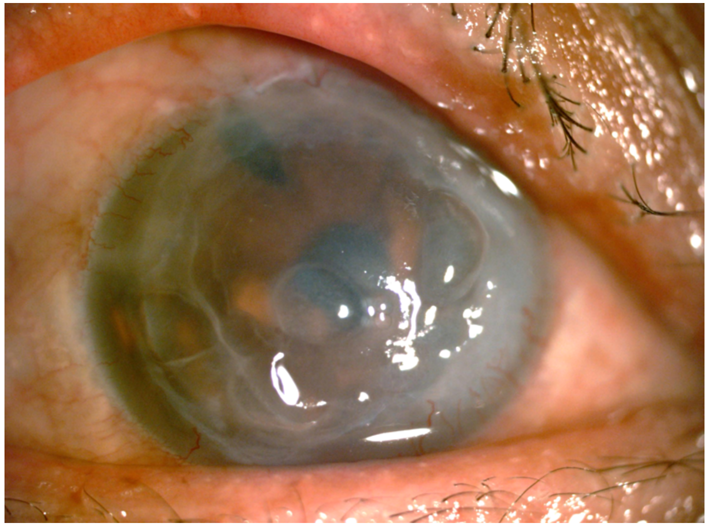	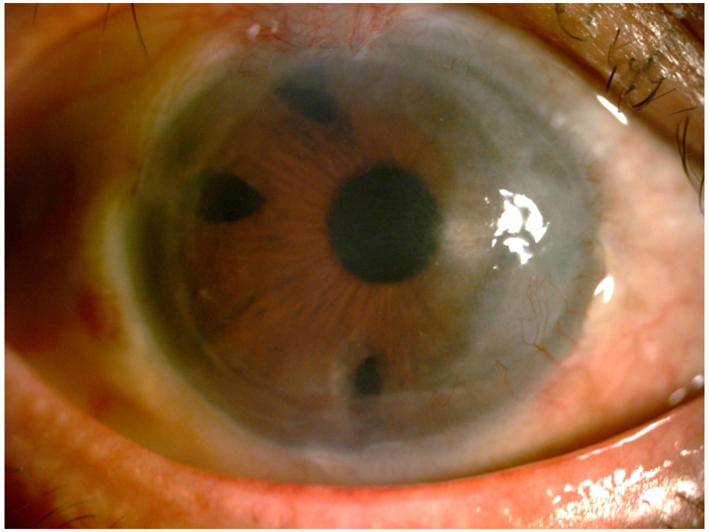

Case	Pre-op	Post-op
09	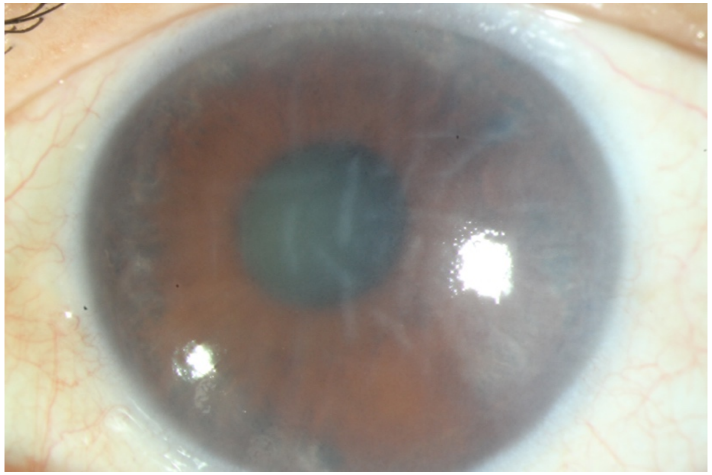	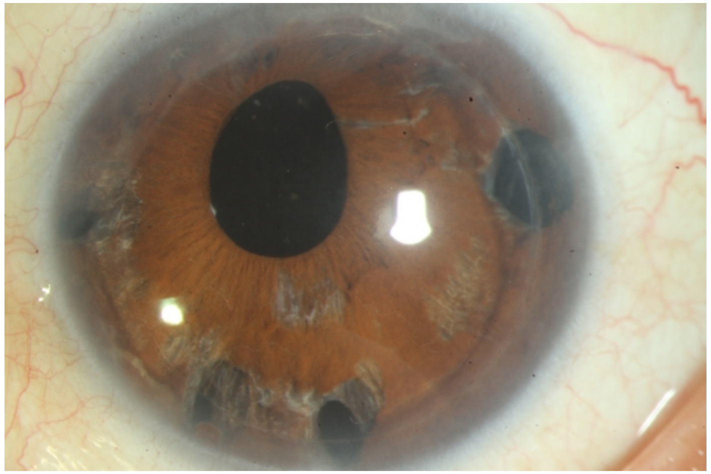
10	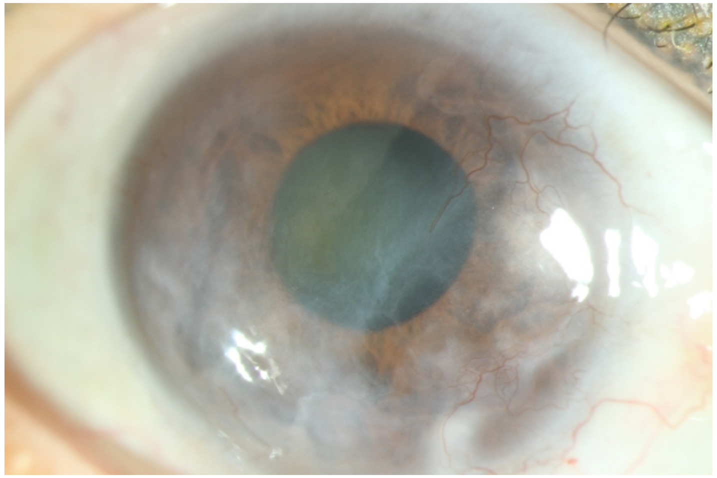	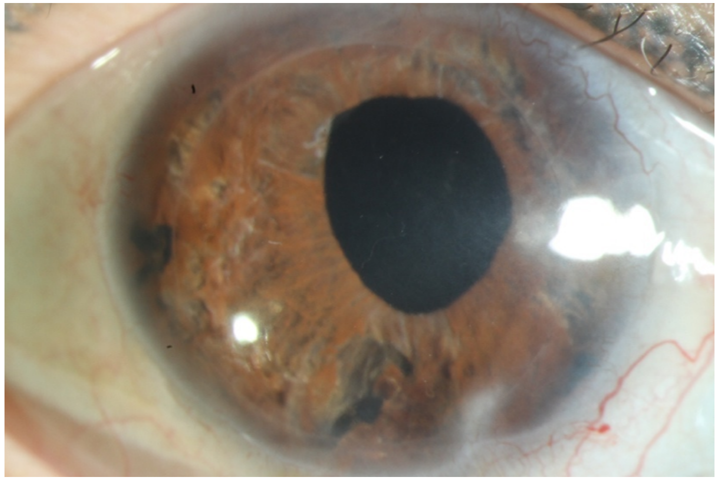

Case	Pre-op	Post-op
11	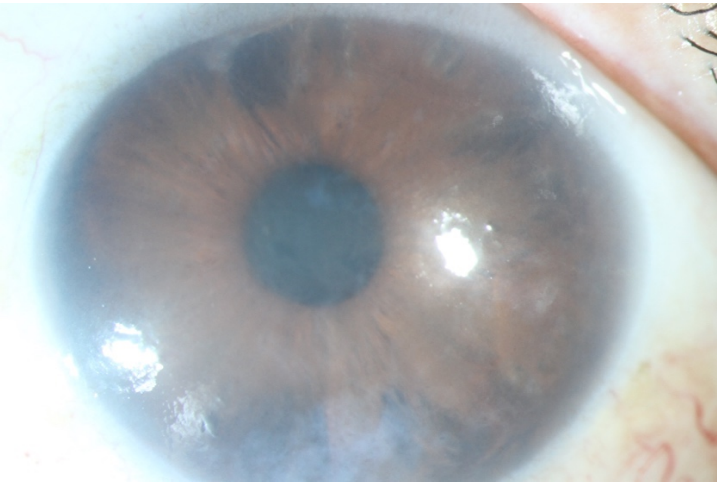	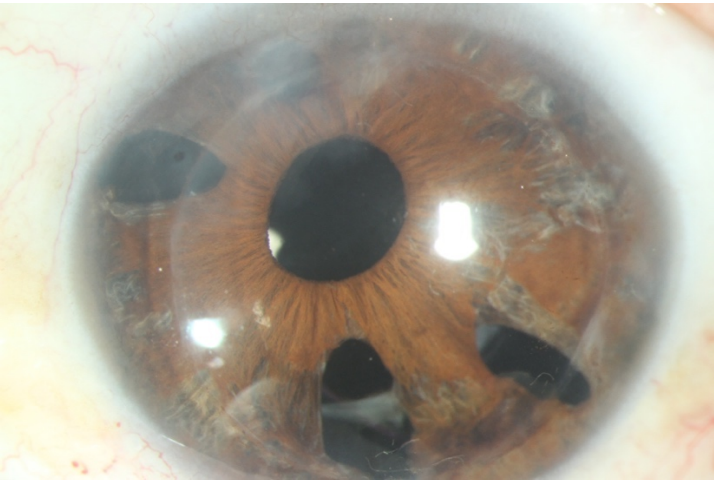

Supplementary Table 1-2. Preoperative and postoperative photographs for each case in SI+EK group. SI, spokewise iridotomy; EK, endothelial keratoplasty; Pre-op, preoperative; Post-op, postoperative.

Preoperative, intraoperative and postoperative details for EK group												
**Case**	**Age/ gender**	**Eye**	**Pre-op BCVA (Snellen)**	**Pre-op clinical findings**	**Prior glaucoma (Y/N)**	**Prior AGMs**	**Prior surgical history**	**Surgical procedures**	**Post-op BCVA (Snellen)**	**Post-op AGMs**	**Post-op events**	**Follow-up (months)**
12	48/F	OD	HM/50cm	Iris root incision at 1 o'clock, corectopia, iris atrophy, PAS at 12 to 3 and 6 to 9 o'clock	Yes	2	Phaco/PCIOL, canaloplasty+ KDB	DSAEK, synechiolysis	20/333	4	Vision doesn't improve due to glaucomatous optic neuropathy. Pupillary block hypertension at the 1-st night and part of air bubble ventilated. Increased IOP could not be controlled by topical medication since 1-month.	2

Case	Age/ gender	Eye	Pre-op BCVA (Snellen)	Pre-op clinical findings	Prior glaucoma (Y/N)	Prior AGMs	Prior surgical history	Surgical procedures	Post-op BCVA (Snellen)	Post-op AGMs	Post-op events	Follow-up (months)
13	73/F	OS	20/400	Iris atrophy, corectopia, PAS at 2 to 10 o'clock, iris root incision at 10 and 12 o'clock	Yes	2	Trab, vitrectomy, epiretinal macular membrane exfoliation, Phaco/PCIOL	DSAEK, synechiolysis	20/333	3	Pupillary block hypertension at the 1-st night and part of air bubble ventilated. Increased IOP could not be controlled by topical medication since 5-month and underwent paracentesis of filtering bleb. Irreversible corneal edema at 10-month.	10
14	50/M	OS	20/60	Iris atrophy, corectopia, broad PAS, iris root incision at 12 and 2 o'clock	Yes	0	EK, phaco/PCIOL, Trab	DSAEK, synechiolysis	20/400	3	Increased IOP could not be controlled by topical medication since 3-month and underwent Trab surgery. Irreversible corneal edema at 6-month.	6

Case	Age/ gender	Eye	Pre-op BCVA (Snellen)	Pre-op clinical findings	Prior glaucoma (Y/N)	Prior AGMs	Prior surgical history	Surgical procedures	Post-op BCVA (Snellen)	Post-op AGMs	Post-op events	Follow-up (months)
15	50/F	OS	20/200	Iris atrophy, corectopia, polycoria, broad PAS	Yes	1	-	DSAEK, synechiolysis	20/400	0	Pupillary block hypertension at the 1-st night and part of air bubble ventilated. Followed up on call since 7-month.	29
16	51/M	OD	20/2000	Iris atrophy, corectopia, PAS at 10 to 4 o'clock, bleb superiorly	No	0	-	DSAEK, synechiolysis, phaco/PCIOL	20/40	0	-	3
17	64/F	OD	HM/15cm	Iris atrophy, corectopia, broad PAS, bleb superiorly, aphakia	No	0	Phaco	DSAEK, synechiolysis	HM/15cm	3	Vision doesn't improve due to aphakia. Endothelial cells could not be identified by confocal microscope at 2-month and irreversible edema at the same month.	6

Case	Age/ gender	Eye	Pre-op BCVA (Snellen)	Pre-op clinical findings	Prior glaucoma (Y/N)	Prior AGMs	Prior surgical history	Surgical procedures	Post-op BCVA (Snellen)	Post-op AGMs	Post-op events	Follow-up (months)
18	45/M	OS	20/333	PAS at 12 o'clock	Yes	1	-	DSAEK, synechiolysis, peripheral iridotomy at 12 o'clock	20/80	2	Pupillary block hypertension at the 1-st night and part of air bubble ventilated. Endothelial cells could not be identified by confocal microscope since 1-month, intravitreal injection of ganciclovir at 1-month , irreversible corneal edema at 6-month.	6
19	39/M	OD	20/400	Iris atrophy, corectopia, broad PAS	Yes	1	EK, phaco/PCIOL, Trab	DSAEK, synechiolysis	20/500	3	Pupillary block hypertension at the 1-st night and part of air bubble ventilated. Irreversible corneal edema at 2-month.	2

Case	Age/ gender	Eye	Pre-op BCVA (Snellen)	Pre-op clinical findings	Prior glaucoma (Y/N)	Prior AGMs	Prior surgical history	Surgical procedures	Post-op BCVA (Snellen)	Post-op AGMs	Post-op events	Follow-up (months)
20	46/M	OD	20/2000	Iris atrophy, corectopia, keratoleukoma, PAS at 4 to 6 o'clock	Yes	2	Phaco	DSAEK, synechiolysis, PCIOL	20/133	1	Vision doesn't improve due to keratoleukoma	3
21	68/F	OD	20/500	Iris atrophy, corectopia	Yes	2	Trab	DSAEK, phaco/PCIOL	20/250	2	-	49
22	61/F	OD	FC/20cm	Iris atrophy, corectopia, PAS at 4 to 7 and 12 o'clock	No	0	Phaco/PCIOL	DSAEK, synechiolysis	20/25	0	Vitrectomy with macular epiretinal membranes peeling at 5-month	59
23	42/M	OD	FC/30cm	Iris atrophy, corectopia, broad PAS, bleb superiorly	No	0	-	DSAEK, synechiolysis	/	2	Pupillary block hypertension at the 1-st night and part of air bubble ventilated.	6

Case	Age/ gender	Eye	Pre-op BCVA (Snellen)	Pre-op clinical findings	Prior glaucoma (Y/N)	Prior AGMs	Prior surgical history	Surgical procedures	Post-op BCVA (Snellen)	Post-op AGMs	Post-op events	Follow-up (months)
24	32/M	OD	20/500	Iris atrophy, corectopia, broad PAS	No	0	-	DSAEK, synechiolysis	20/33	1	-	57
25	67/M	OD	20/50	Iris atrophy, corectopia, broad PAS, bleb superiorly	No	0	-	DSAEK, synechiolysis, phaco/PCIOL	20/66	0	Pupillary block hypertension at the 1-st night and part of air bubble ventilated. Followed up on call since 5-month.	67
26	62/F	OD	20/250	Iris atrophy, corectopia, broad PAS, bleb superiorly	No	0	-	DSAEK, synechiolysis, phaco/PCIOL	20/40	0	Pupillary block hypertension at the 1-st night and part of air bubble ventilated.	9
27	47/F	OS	20/250	Iris atrophy, broad PAS	No	0	--	DSAEK, synechiolysis, phaco/PCIOL	20/50	0	Pupillary block hypertension at the 1-st night and part of air bubble ventilated.	1

Case	Age/ gender	Eye	Pre-op BCVA (Snellen)	Pre-op clinical findings	Prior glaucoma (Y/N)	Prior AGMs	Prior surgical history	Surgical procedures	Post-op BCVA (Snellen)	Post-op AGMs	Post-op events	Follow-up (months)
28	49/F	OS	20/200	Iris atrophy, corectopia, broad PAS	No	0	-	DSAEK, synchiolysis, phaco/PCIOL	HM/15cm	3	Increased IOP could not be controlled by topical medication since 2-month, underwent cyclophotocoagulation at 2-month	5
29	42/M	OS	FC/15cm	Broad PAS, bleb superiorly	No	0	-	DSAEK, synchiolysis, peripheral iridotomy at 11 o'clock	/	1	Pupillary block hypertension at the 1-st night and part of air bubble ventilated.	1
30	51/F	OS	HM/15cm	Iris atrophy, corectopia, broad PAS	Yes	3	EK, phaco/PCIOL	DSAEK, synchiolysis	HM/15cm	2	Vision doesn't improve due to glaucomatous optic neuropathy.	54
31	63/M	OS	20/166	Iris atrophy, broad PAS	Yes	0	Anti-glaucoma surgery(type unknown)	DSAEK, phaco/PCIOL, synechiolysis	20/33	0	-	1

Supplementary Table 1-3. Preoperative, intraoperative and postoperative details for each case in EK group. EK, endothelial keratoplasty; Pre-op, preoperative; Post-op, postoperative; BCVA, best corrected visual acuity; AGM, antiglaucoma medication; PAS, peripheral anterior

synechiae; Trab, trabeculectomy; PCIOL, posterior chamber intraocular lens; DSAEK, descemet stripping automated endothelial keratoplasty; IOP, intraocular pressure; GDD, glaucoma drainage device.

Preoperative and postoperative photographs for EK group		
**Case**	**Pre-op**	**Post-op**
12	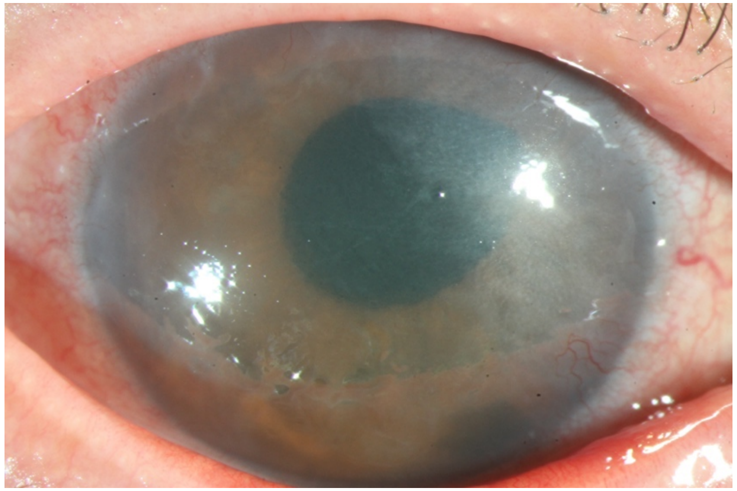	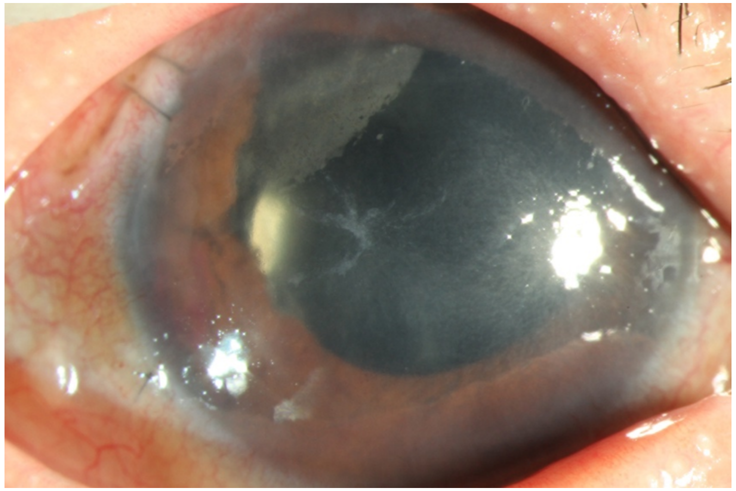
13	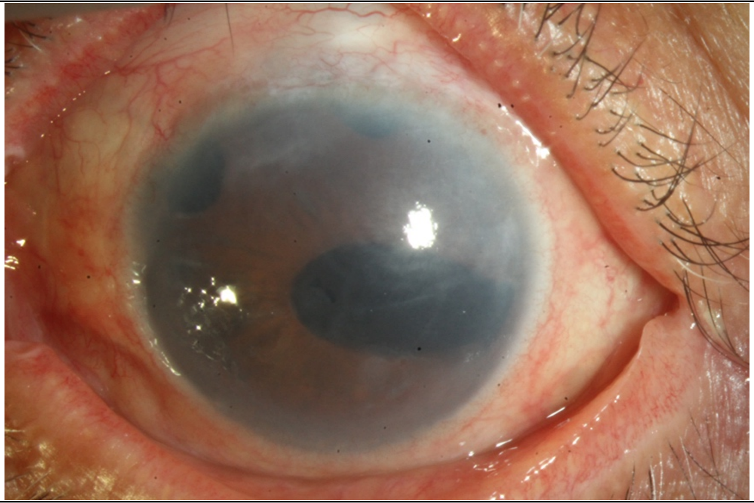	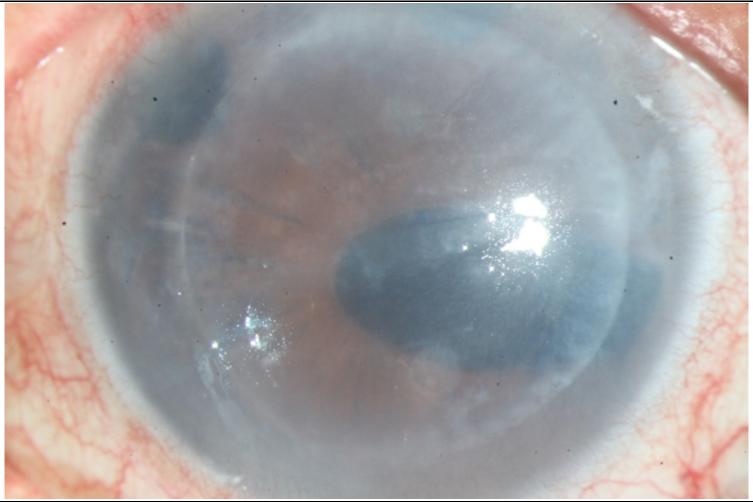

Case	Pre-op	Post-op
14	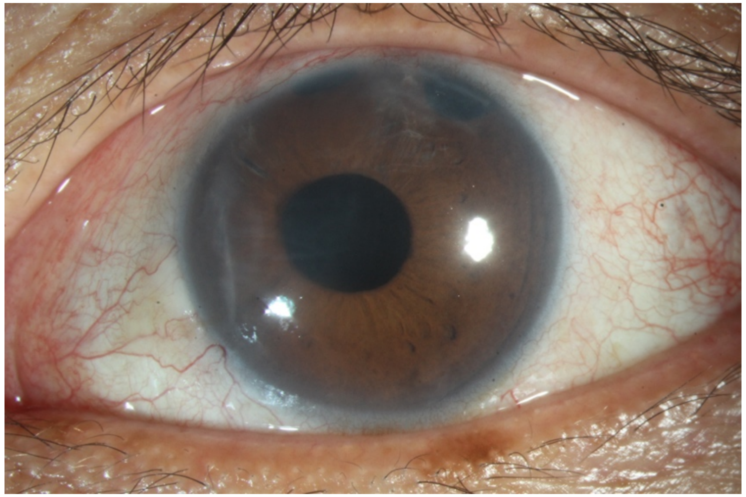	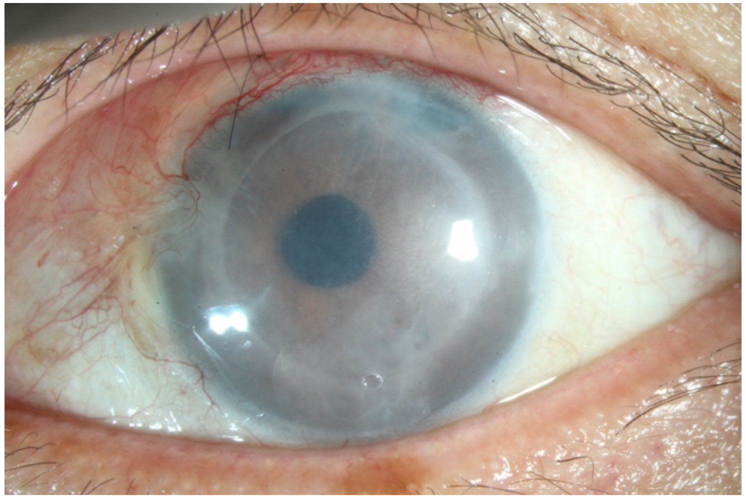
15	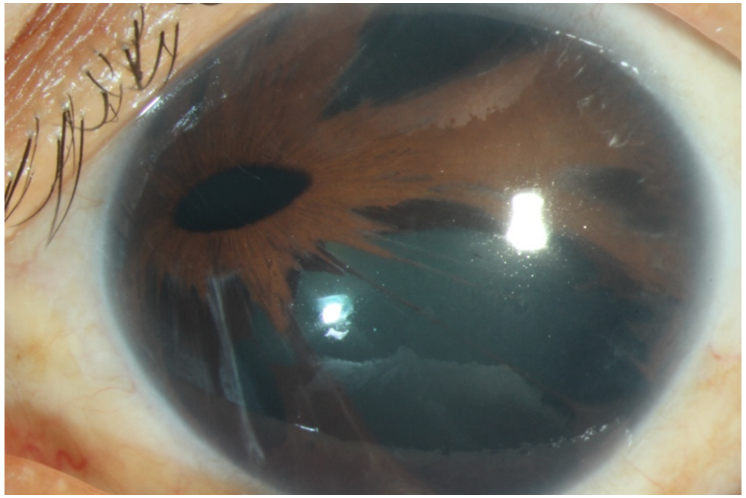	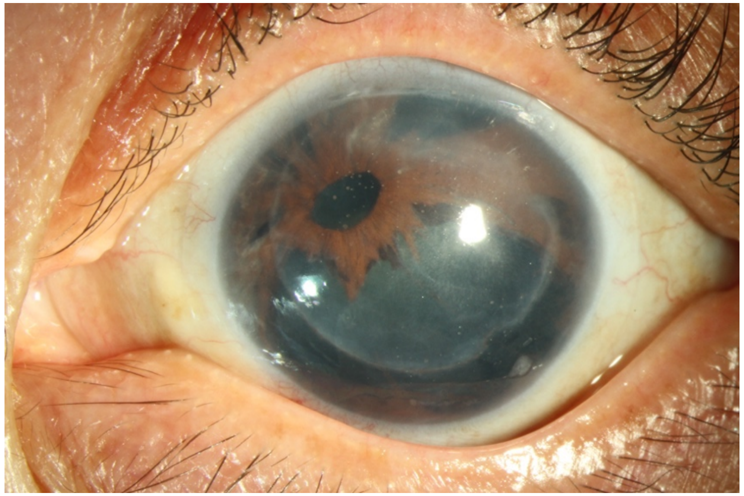

Case	Pre-op	Post-op
16	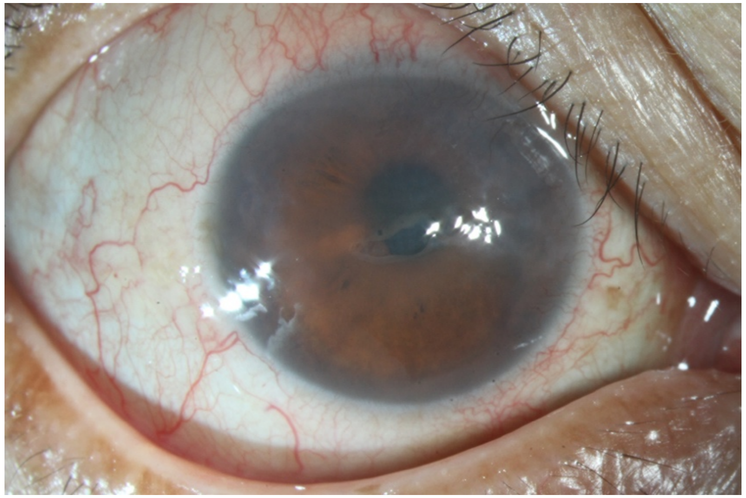	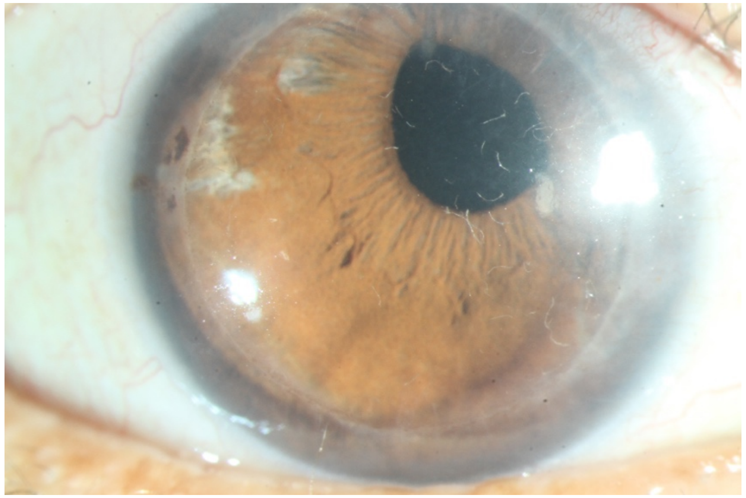
17	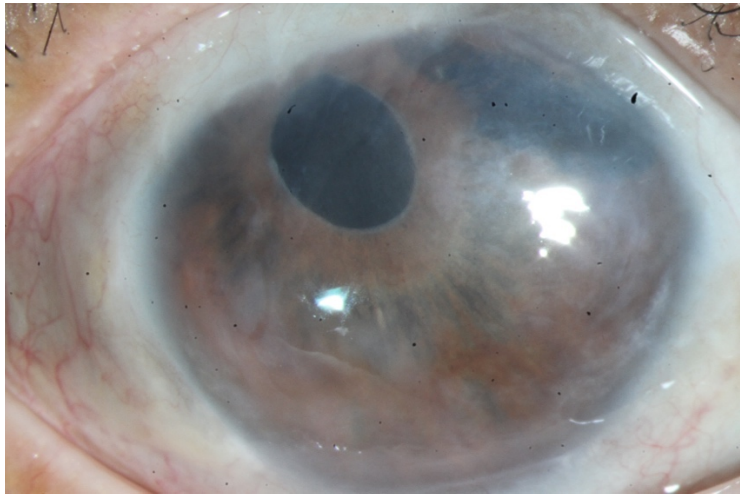	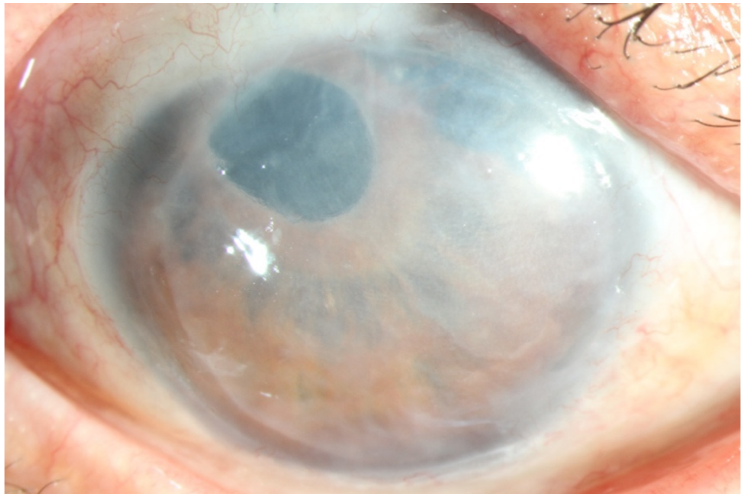

Case	Pre-op	Post-op
18	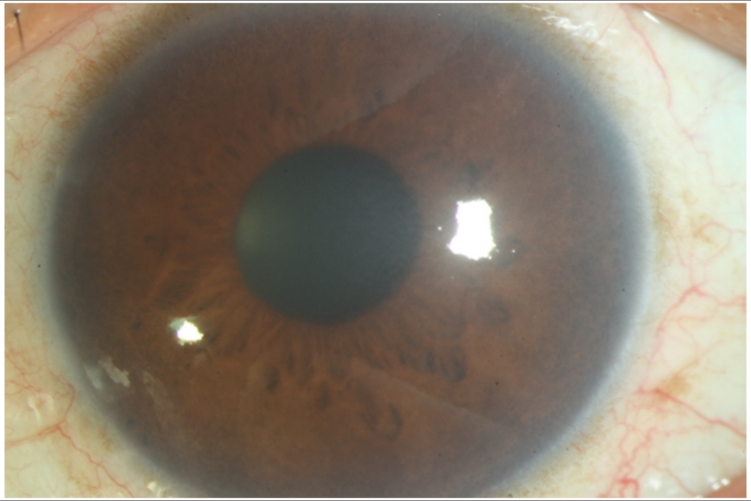	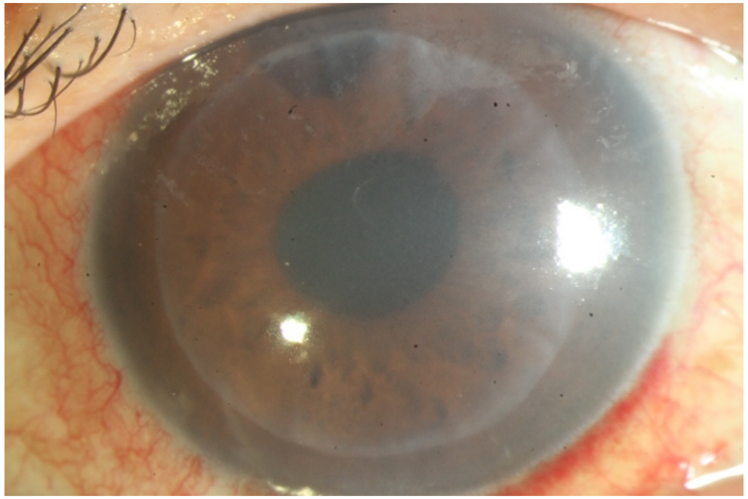
19	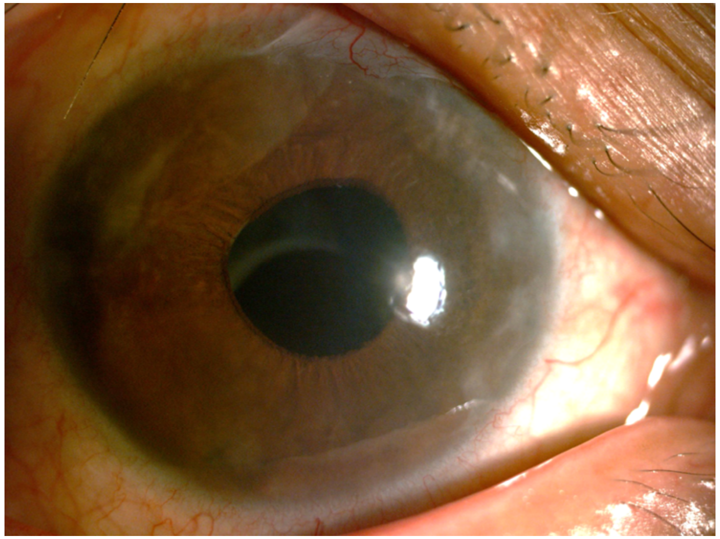	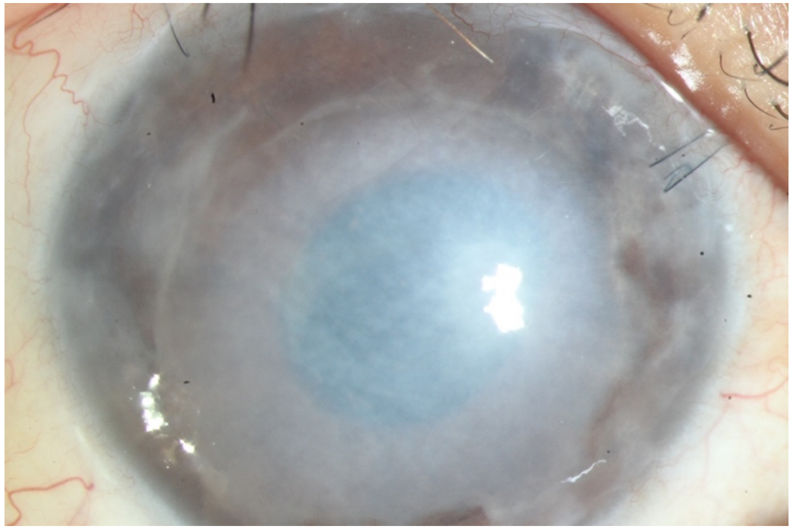

Case	Pre-op	Post-op
20	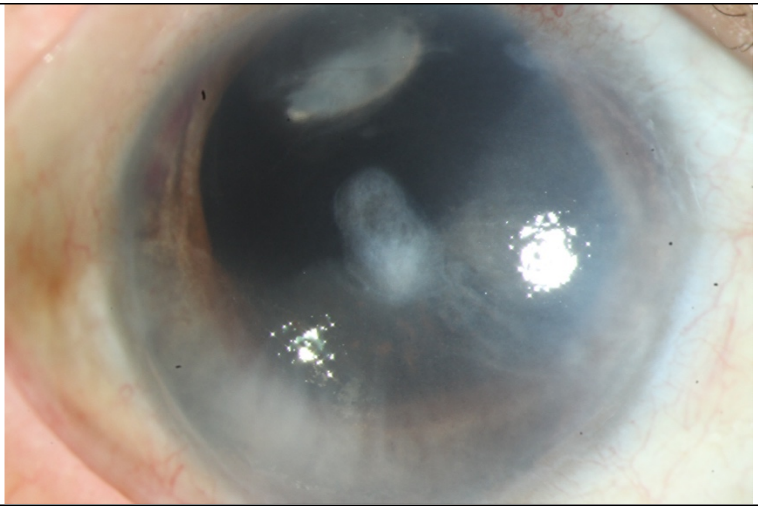	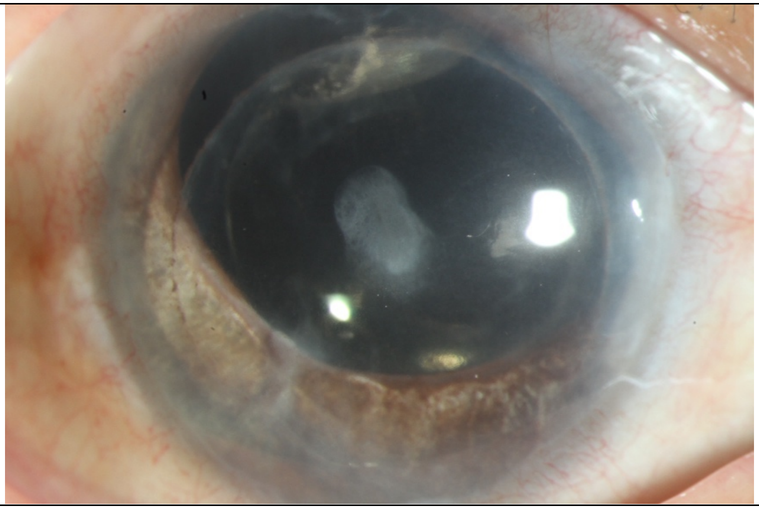
21	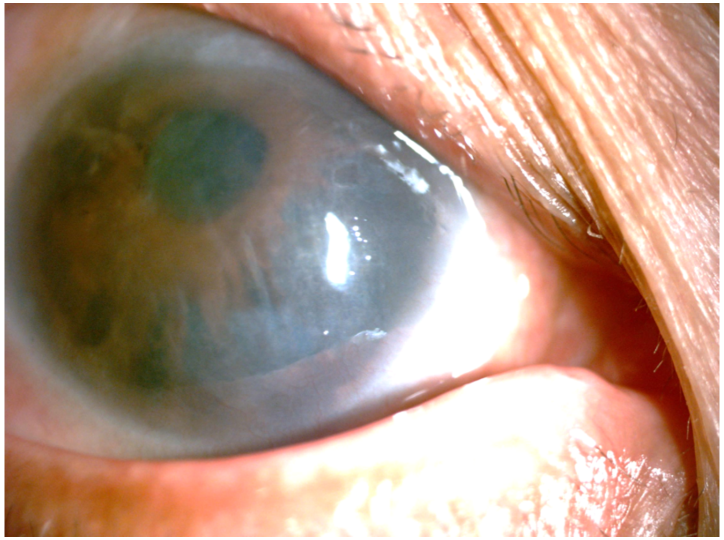	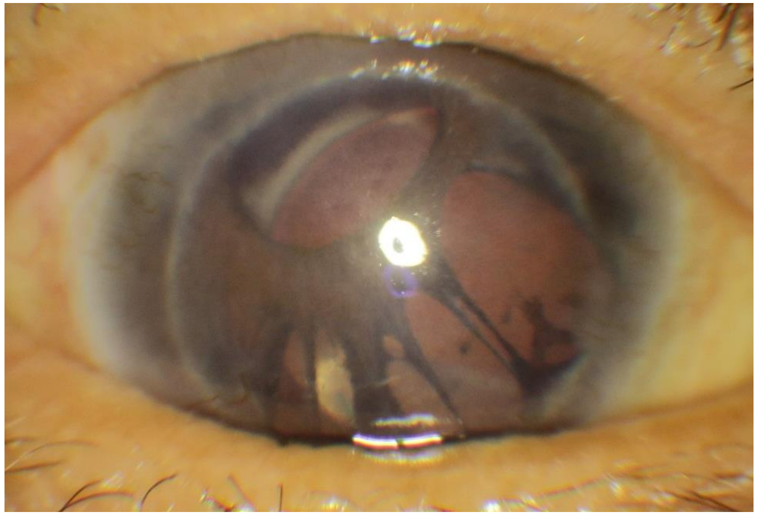

Case	Pre-op	Post-op
22	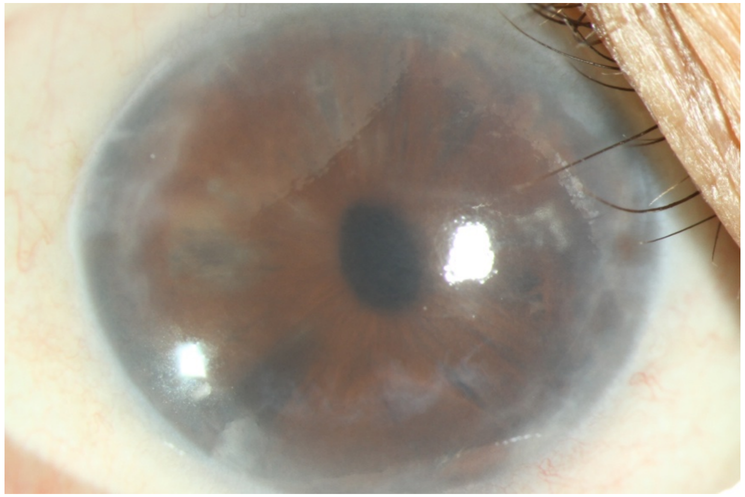	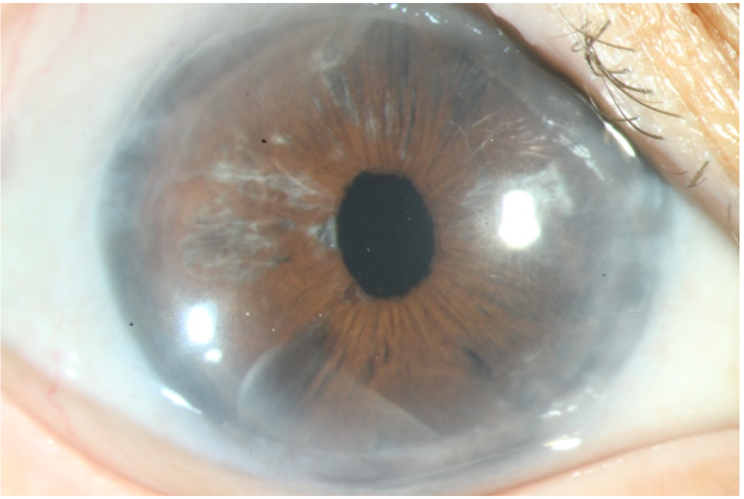
23	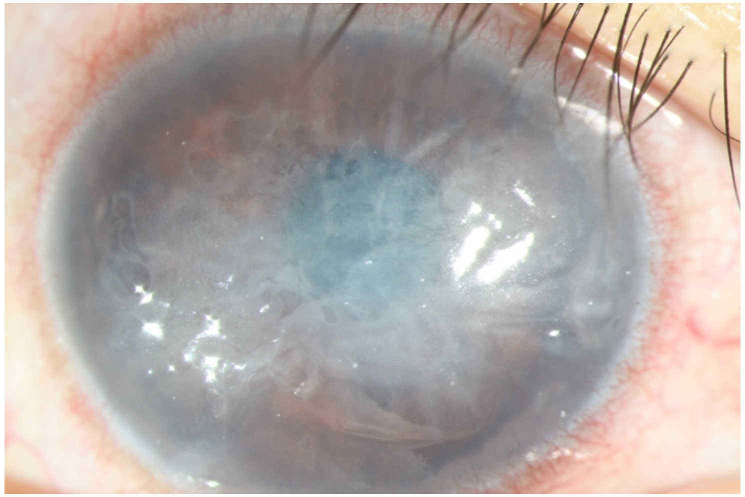	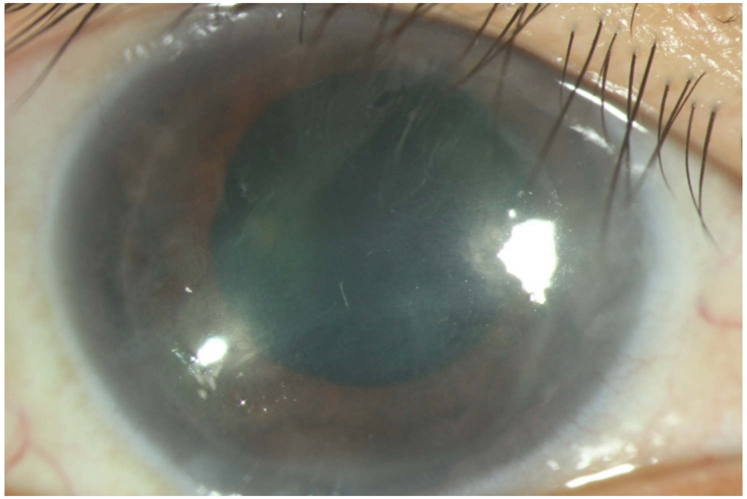

Case	Pre-op	Post-op
24	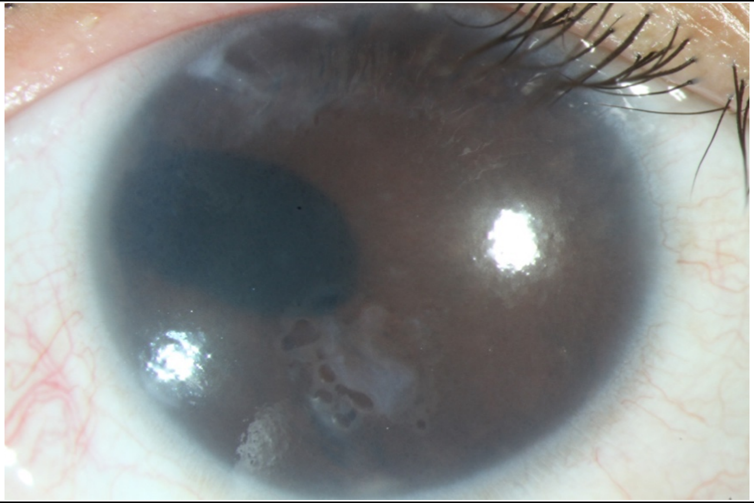	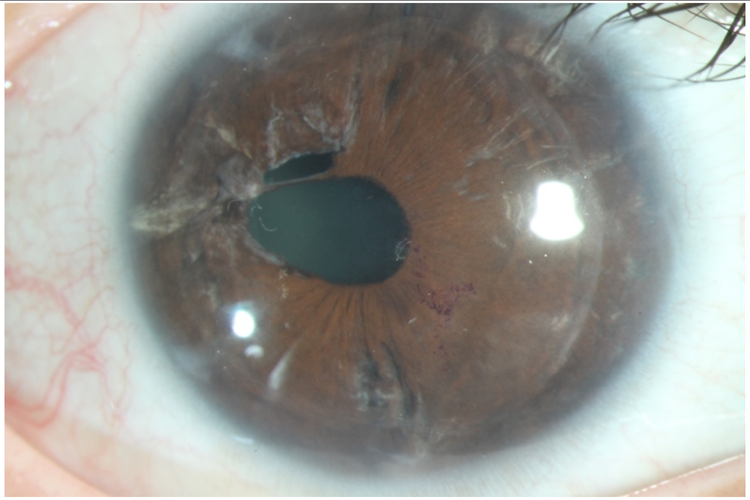
25	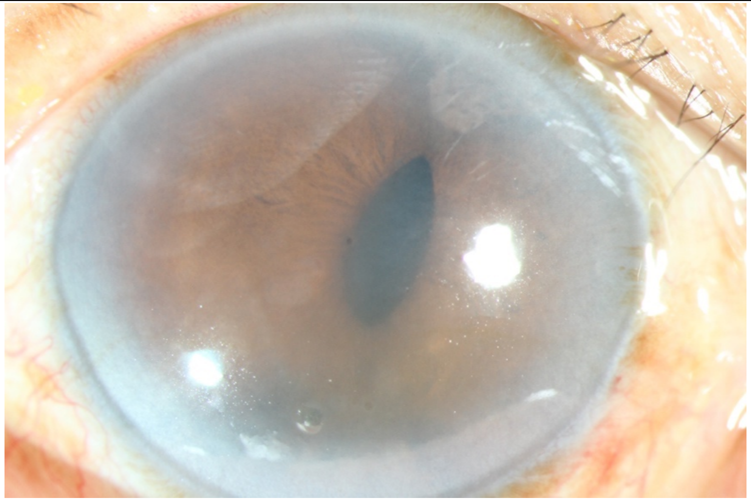	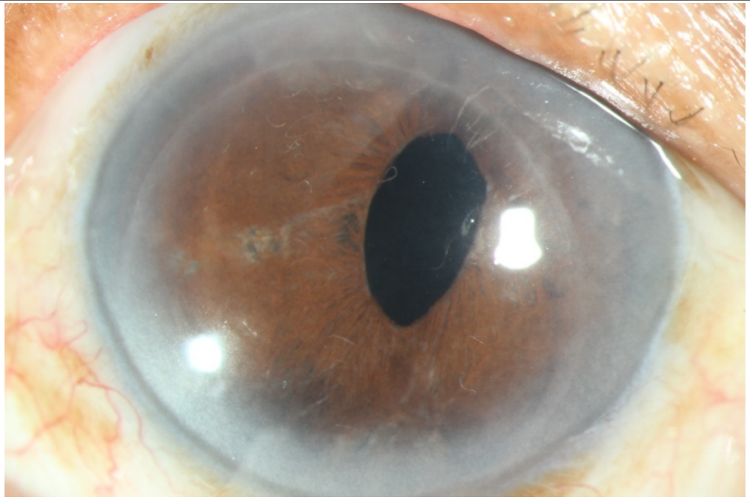

Case	Pre-op	Post-op
26	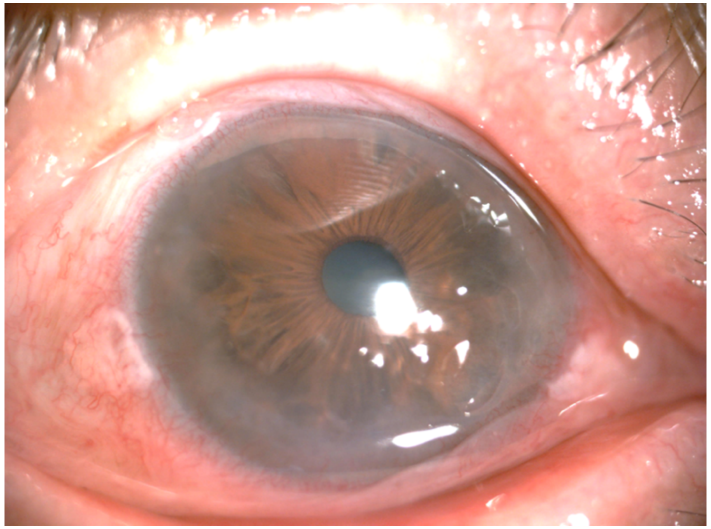	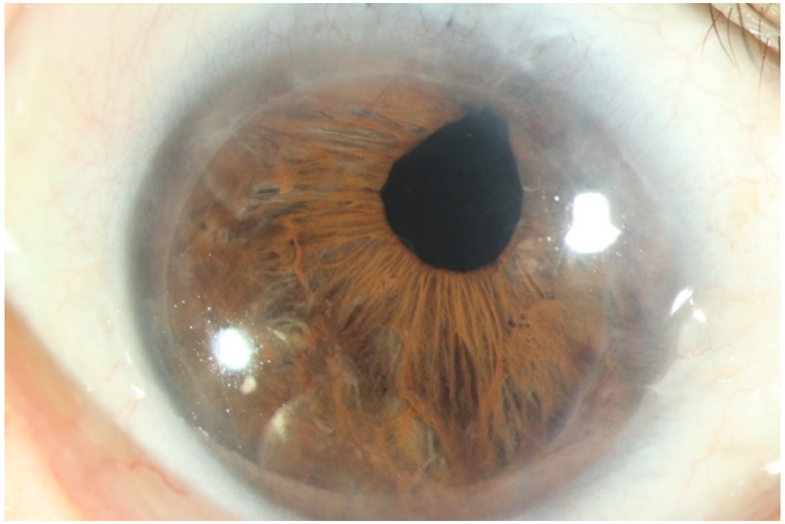
27	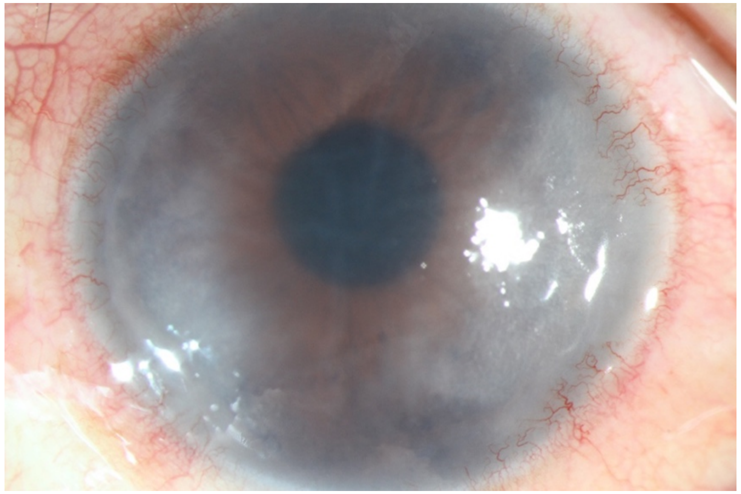	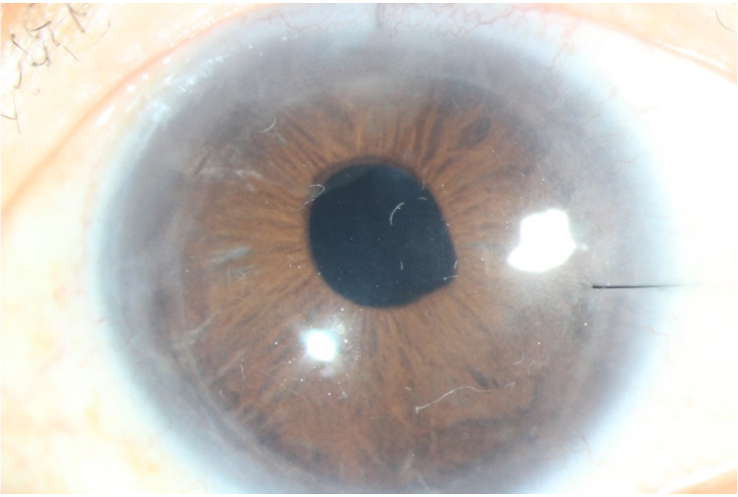

Case	Pre-op	Post-op
28	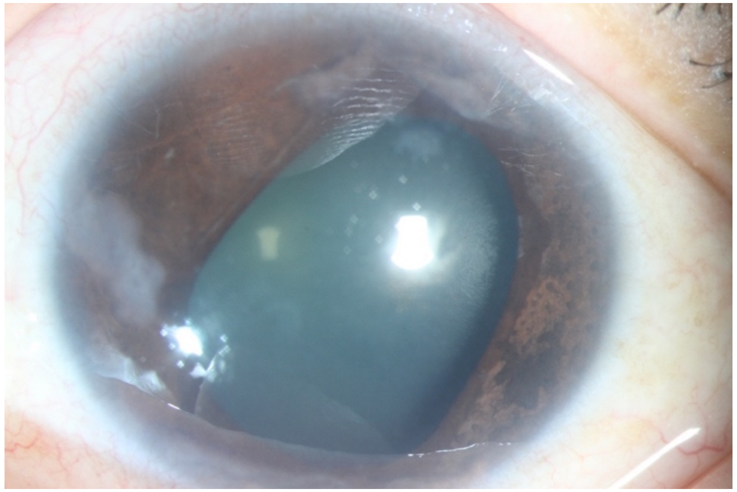	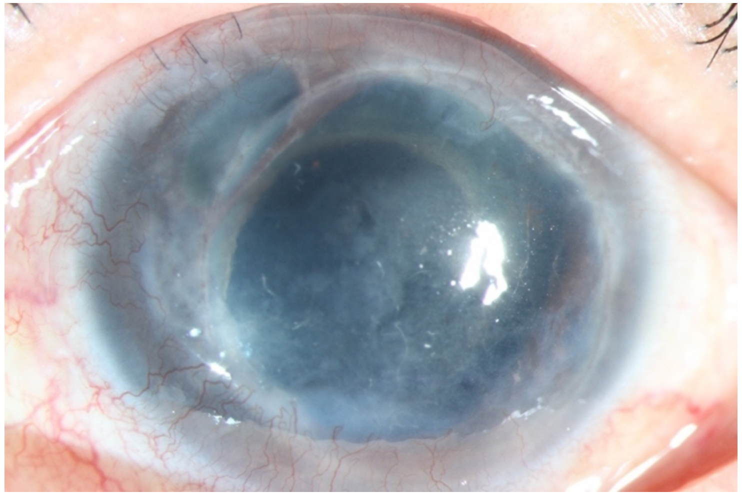
29	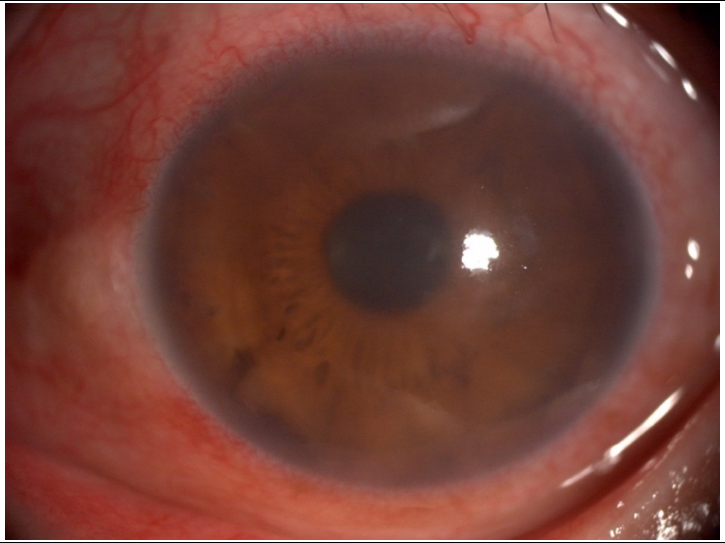	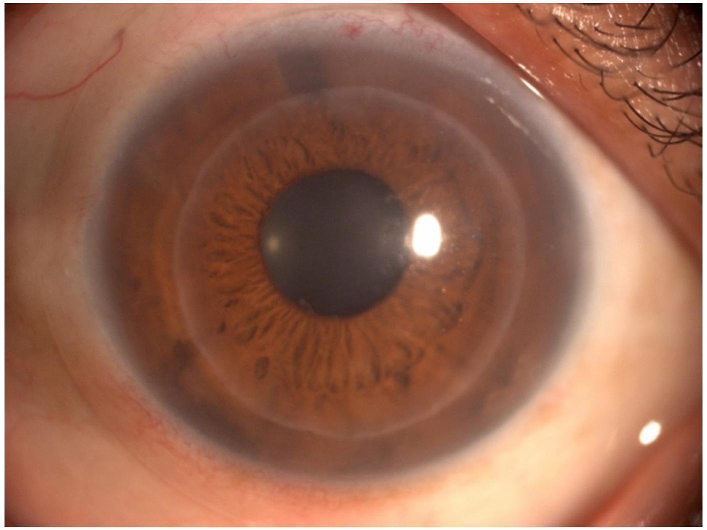

Case	Pre-op	Post-op
30	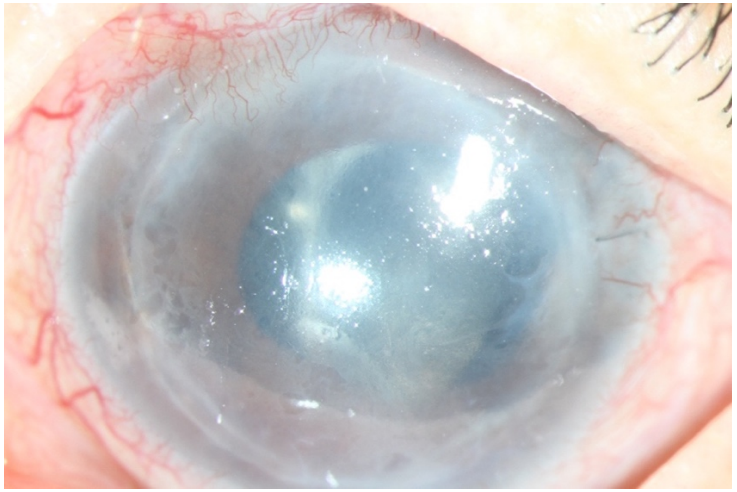	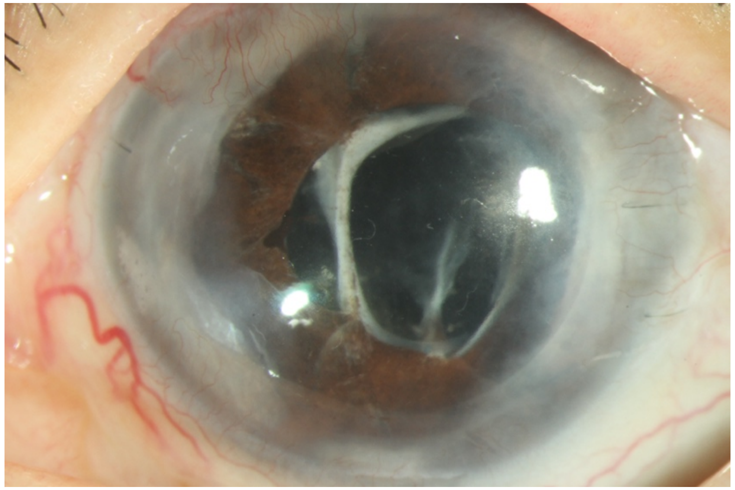
31	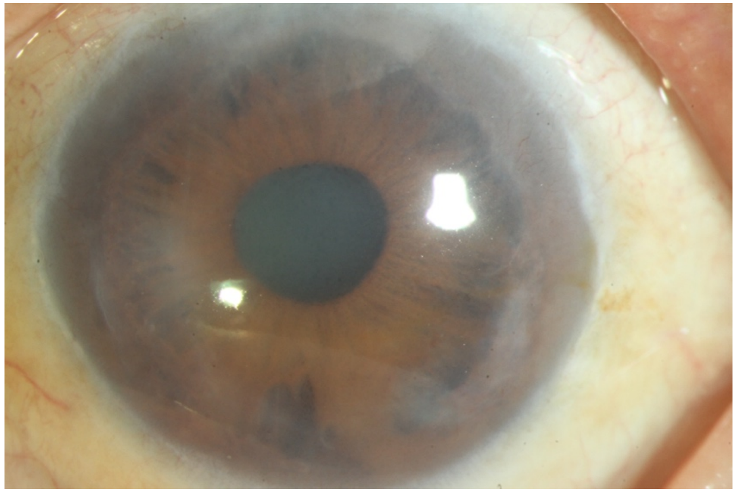	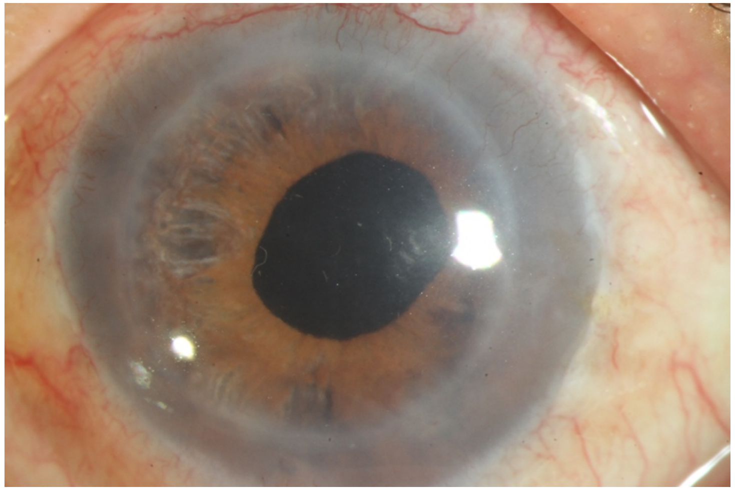

Supplementary Table 1-4. Preoperative, and postoperative photographs for each case in EK group. EK, endothelial keratoplasty; Pre-op, preoperative; Post-op, postoperative.

Supplementary Table 2. Demographic and medical history of different surgical groups (n=31)

	SI+EK (n=11)	EK (n=20)	P-value
Age (year)	56.09±13.61	52.50±10.95	0.429
Follow-up time (month)	31.89±24.76	6.17 (1.72, 44.00)	0.117
Gender (n, %)			0.707
male	7 (63.6%)	10 (50.0%)	
female	4 (36.4%)	10 (50.0%)	
Eye (n, %)			0.636
OD	6 (54.5%)	11 (55.0%)	
OS	5 (45.5%)	9 (45.0%)	
Glaucoma history (n, %)			0.128
Yes	9 (81.8%)	10 (50.0%)	
No	2 (18.2%)	10 (50.0%)	
Keratoplasty history(n, %)			0.210
Yes	4 (36.4%)	3 (15.0%)	
No	7 (63.6%)	17 (85.0%)	

Cataract surgery history (n, %)			0.698
Yes	3 (27.3%)	8 (40.0%)	
No	8 (72.7%)	12 (60.0%)	
Antiglaucoma surgery history (n, %)			0.452
Yes	5 (45.5%)	6 (30.0%)	
No	6 (54.5%)	14 (70.0%)	

SI, spokewise iridotomy; EK, endothelial keratoplasty

Supplementary Table 3. Preoperative and surgical details in the different surgical groups (n=31)

	SI+EK (n=11)	EK (n=20)	P-value
Pre-op AGMs (n)	0 (0, 2)	0 (0, 1.75)	0.763
Pre-op IOP (mmHg)	18.00 (15.10, 20.00)	16.00 (12.50, 21.75)	0.482
Pre-op BCVA [a]	1.43±0.46	1.23±0.48	0.314
Pre-op ECD (cells/mm^2)[b]	0 (0, 822)	628.5 (435.25, 906.5)	0.225
Pre-op CCT (um)[c]	740 (660, 790)	665 (587.5, 810)	0.139
Pre-op PAS range (n)[d]	12 (11, 12)	9 (7.25, 12)	0.088
Donor ECD (cells/mm^2)	3053 (3000, 3333)	3097.50 (3000, 3315.75)	0.983
Graft size (mm)	8.00 (7.50, 8.00)	8.00 (8.00, 8.00)	0.678
Combine cataract surgery (n, %)			1.000
Yes	5 (45.5%)	8 (40.0%)	
No	6 (54.5%)	12 (60.0%)	
Peripheral iridotomy number (n)[e]	4.50（3.00，6.75）	-	-
Maximum interval of iris incision (n)	3.05±1.97	-	-
Surgeon (n, %)			0.060
Surgeon 1	9 (81.8%)	9 (45.0%)	

Surgeon 2	1 (9.1%)	9 (45.0%)
Surgeon 3	1 (9.1%)	0
Surgeon 4	0	2 (10.0%)

SI, spokewise iridotomy; EK, endothelial keratoplasty; Pre-op, preoperative; AGMs, antiglaucoma medications; IOP, intraocular pressure； BCVA, best corrected visual acuity; ECD, endothelial cell density; CCT, central corneal thickness; PAS, peripheral anterior synechiae.

a: 6 eyes with other vision-affecting causes were excluded (2 in SI+EK group, 4 in EK group). b: 7 in SI+EK group and 16 in EK group has available data, unclear ECD are counted as 0; c: 11 in SI+EK group and 14 in EK group has available data; d: 11 in SI+EK group and 16 in EK group has available data; e: calculate after exclude case No.2 (Total iridectomy).

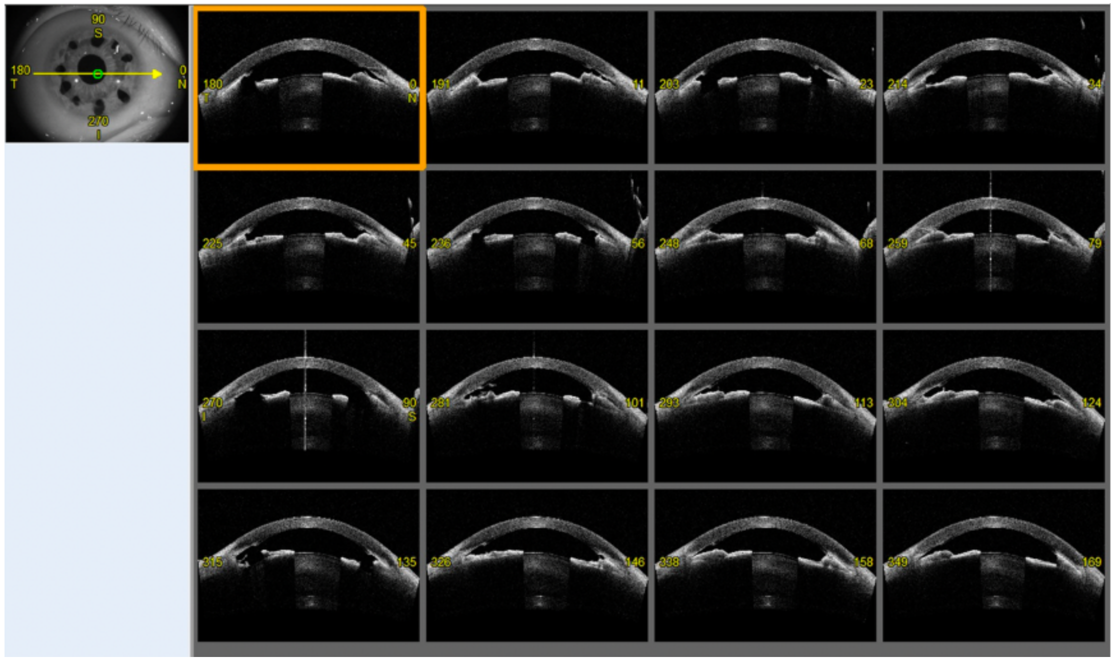

Supplementary Figure 1 An example of postoperative PAS in patients in the SI+EK group

The AS-OCT result of patient NO.7 30 months after the operation showed that although PAS occurred, the degree of PAS was mild at the location with iris root incision, and it did not affect the flow of aqueous humor, so only the positions without iris incision were recorded.

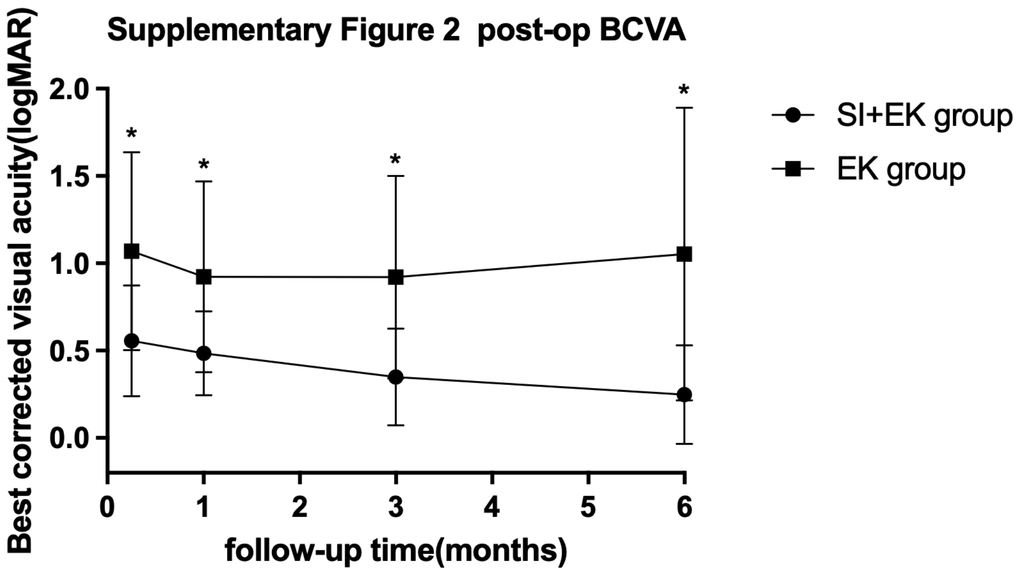

Supplementary Figure 2. Follow-up data of BCVA

BCVA is better in the SI+EK group, at 1 week, 1 month, 3 months, and 6 months after the operation, the BCVA of the two groups were 0.56±0.32 vs 1.07±0.57, 0.48±0.24 vs 0.92±0.55, 0.35±0.28 vs 0.92±0.58, 0.10(IQR 0.10-0.40) vs 0.91(IQR 0.23-1.85), respectively, with all $p<0.05$.
